# Genome co-amplification upregulates a mitotic gene network activity that predicts outcome and response to mitotic protein inhibitors in breast cancer

**DOI:** 10.1186/s13058-016-0728-y

**Published:** 2016-07-01

**Authors:** Zhi Hu, Jian-Hua Mao, Christina Curtis, Ge Huang, Shenda Gu, Laura Heiser, Marc E. Lenburg, James E. Korkola, Nora Bayani, Shamith Samarajiwa, Jose A. Seoane, Mark A. Dane, Amanda Esch, Heidi S. Feiler, Nicholas J. Wang, Mary Ann Hardwicke, Sylvie Laquerre, Jeff Jackson, Kenneth W. Wood, Barbara Weber, Paul T. Spellman, Samuel Aparicio, Richard Wooster, Carlos Caldas, Joe W. Gray

**Affiliations:** Department of Biomedical Engineering, School of Medicine, Oregon Health & Science University, 3303 SW Bond Ave., CH13B, Portland, OR 97239 USA; Life Sciences Division, Lawrence Berkeley National Laboratory, Berkeley, CA 94127 USA; Department of Medicine, Division of Oncology and Department of Genetics, Stanford University School of Medicine, Stanford, CA 94305 USA; Department of Pathology and Laboratory Medicine, Boston University School of Medicine, Boston, MA 02215 USA; MRC Cancer Unit, University of Cambridge, Cambridge, CB2 0XZ UK; GlaxoSmithKline, Collegeville, PA 19425 USA; Cytokinetics, Inc., South San Francisco, CA 94080 USA; Molecular Oncology, BC Cancer Research Centre, Vancouver, Canada; Cancer Research UK, Cambridge Institute, Cambridge, UK

**Keywords:** Breast cancer, Mitotic index, Predictive biomarker, Novel therapeutics

## Abstract

**Background:**

High mitotic activity is associated with the genesis and progression of many cancers. Small molecule inhibitors of mitotic apparatus proteins are now being developed and evaluated clinically as anticancer agents. With clinical trials of several of these experimental compounds underway, it is important to understand the molecular mechanisms that determine high mitotic activity, identify tumor subtypes that carry molecular aberrations that confer high mitotic activity, and to develop molecular markers that distinguish which tumors will be most responsive to mitotic apparatus inhibitors.

**Methods:**

We identified a coordinately regulated mitotic apparatus network by analyzing gene expression profiles for 53 malignant and non-malignant human breast cancer cell lines and two separate primary breast tumor datasets. We defined the mitotic network activity index (MNAI) as the sum of the transcriptional levels of the 54 coordinately regulated mitotic apparatus genes. The effect of those genes on cell growth was evaluated by small interfering RNA (siRNA).

**Results:**

High MNAI was enriched in basal-like breast tumors and was associated with reduced survival duration and preferential sensitivity to inhibitors of the mitotic apparatus proteins, polo-like kinase, centromere associated protein E and aurora kinase designated GSK462364, GSK923295 and GSK1070916, respectively. Co-amplification of regions of chromosomes 8q24, 10p15-p12, 12p13, and 17q24-q25 was associated with the transcriptional upregulation of this network of 54 mitotic apparatus genes, and we identify transcription factors that localize to these regions and putatively regulate mitotic activity. Knockdown of the mitotic network by siRNA identified 22 genes that might be considered as additional therapeutic targets for this clinically relevant patient subgroup.

**Conclusions:**

We define a molecular signature which may guide therapeutic approaches for tumors with high mitotic network activity.

**Electronic supplementary material:**

The online version of this article (doi:10.1186/s13058-016-0728-y) contains supplementary material, which is available to authorized users.

## Background

Studies of the molecular biology of cell division have revealed an intricate network of structural proteins, molecular motors, regulatory kinases and phosphatases that are required for error-free chromosome segregation. Upregulation of the genes that encode these proteins, the ensemble of which is referred to hereafter as the mitotic apparatus network, is associated with genome instability [1], carcinogenesis [2–4], and reduced survival duration [5]. As high mitotic activity is associated with the genesis and progression of many cancers, small molecule inhibitors of mitotic apparatus proteins are now being developed and evaluated clinically as anticancer agents [5–10]. Proteins currently being targeted include the polo-like kinase 1 (PLK1 [5]), the aurora kinases (AURKA) [11] and (AURKB/C) [2, 12], centromere associated protein E (CENPE) [13], and the kinesin spindle protein [14]. With clinical trials of several of these experimental compounds commencing, it is important to understand the molecular mechanisms that determine high mitotic activity, identify tumor subtypes that harbor molecular aberrations that confer high mitotic activity, and to develop molecular markers that define tumors that will be most responsive to mitotic apparatus inhibitors. It is also important to understand how mitotic apparatus protein inhibitors interact in order to guide combined therapeutic strategies.

In a study of transcriptional networks in skin samples from *M. musculus* x *M. spretus* backcross mice, Quigley et al. demonstrated that transcription of a network of mitotic apparatus genes is influenced by germline polymorphisms [15]. As germline polymorphisms associated with aspects of cancer genesis and/or progression are sometimes enhanced in tumors by selection of genomic aberrations that further alter transcription of the target genes [15–17], we investigated the possibility that the high mitotic network activity characteristic of aggressive breast cancer is influenced by genomic aberrations that accumulate during breast cancer genesis and progression. Here we show that co-amplification of transcription factors that putatively target mitotic apparatus network genes is strongly associated with increased transcriptional activity of the mitotic apparatus network. We also show that breast cancer cell lines with high mitotic activity are preferentially sensitive to small molecule inhibitors that target mitotic apparatus proteins PLK1, CENPE and AURKB/C, designated GSK462364 [18, 19], GSK923295 [13, 20], and GSK1070916 [21, 22], respectively. Finally, we identify additional candidate mitotic apparatus network targets and suggest strategies to combine inhibitors to counter the development of resistance.

## Methods

### Cell culture

The cell lines described in this study derived from 49 malignant and 4 non-malignant breast tissues and growth conditions for the cell lines have been reported previously [23].

### Experimental compounds

The small-molecule inhibitors GSK1070916, GSK462364, and GSK923295 were provided by GlaxoSmithKline Inc. GSK462364 is a PLK inhibitor and is selective for PLK1 over PLK2 and PLK3 with K_i_^app^ of 0.5 nM, 850 nM, and 1000 nM, respectively. GSK462364 has at least 1000-fold selectivity for PLK1 compared to 48 other protein kinases [19]. GSK1070916 is an ATP competitive inhibitor that is selective for Aurora B and C with K_i_s of 0.38 and 1.5 nM, respectively, and 250-fold selectivity over Aurora A [22]. GSK923295 is an allosteric inhibitor of CENPE with a K_i_ of 3.2 nM. GSK923295 does not compete with either ATP or microtubules and is highly selective for CENPE compared to seven other kinesins [13]. Stock solutions were made at a concentration of 10 mM in dimethyl sulfoxide (DMSO) and stored at −20 °C. Compounds were diluted (1:5 serial dilution) to produce test inhibitor concentrations ranging from 0.0758 nM to 30 μM.

### Cell viability/growth assay and dose response (50 % growth inhibition (GI_50_))

Dose-response curves were determined according to the National Cancer Institute NIH guidelines. In brief, cell suspensions were aliquoted into 96-well plates in 100 μl growth media. Inoculates were incubated for 24 hours at 37 °C for stabilization and then treated with nine doses in triplicate for 72 hours. Cell proliferation was measured with CellTiter-Glo® Luminescent Cell Viability Assay (Promega, Madison, WI, USA). Luminescence was plotted after subtraction of the baseline (an estimate of the number of the cells at time 0). Total growth inhibition doses and 50 % growth inhibition (GI_50_) doses were calculated by GraphPad Prism4 software (GraphPad Software, Inc., La Jolla, CA, USA).

### Datasets

The mitotic gene transcriptional network was assessed in several published microarray data sets profiled with Affymetrix GeneChip arrays (HG-U133A or HG-U133 Plus 2.0). These data include breast cancer [GEO:GSE2034, GEO:GSE1456, and GEO:GSE4922], lung cancer [GEO:GSE3141], ovarian cancer [GEO:GSE9891], Wilms’tumor [GEO:GSE10320], prostate cancer [GEO:GSE8128], glioma [GEO:GSE13041], acute lymphoblastic leukemia [GEO:GSE12995], acute myelogenous leukemia [GEO:GSE12417], and lymphoblast cell lines [GEO:GSE11582]. Mitotic network activity was also examined in various normal tissues [GEO:GSE7307], including normal breast tissue [GEO:GSE10780]. The relationship between MNAI and survival among patients with breast cancer was examined in four datasets (dataset 1: ArrayExpress (http://www.ebi.ac.uk/arrayexpress/) with accession number E-TABM-158; dataset 2: GSE2034; dataset 3: GSE1456 and dataset 4: GSE4922). Data were pre-processed as described in the original publications.

An additional breast cancer dataset (defined as Curtis dataset) consisting of 1980 fresh-frozen primary breast tumors, recently described by Curtis et al. was employed for validation of the mitotic network gene signature and associations between copy number and expression. For all cases, the genome-wide copy number was assessed on the Affymetrix single nucleotide polymorphism (SNP) 6.0 platform and matched RNA was hybridized to Illumina HT-12 bead arrays for gene expression analysis (https://www.ebi.ac.uk/ega/studies/EGAS00000000083). The dimensionality of the copy number data was reduced by merging regions with similar profiles across all samples based on the CGH regions algorithm [24], resulting in 2995 regions. The mitotic network activity index (MNAI) was computed by utilizing probes with a perfect transcriptomic match based on reannotation of the Illumina platform [25]. Averages were taken when multiple “perfect” or “good” probes were present on the array. Probes annotated as “bad” were excluded from the analysis, except when they were the only probe available for a particular mitotic network gene (*DEPDC1*, *GTSE1*). Samples were classified into the intrinsic subtypes based on PAM50 [26] and the integrative clusters (IC) as defined by Curtis et al [27].

### Statistical analysis

The correlation among the cellular GI_50_ values of GSK462364, GSK1070916 and GSK92325 was assessed with the Pearson correlation test. Tumor expression profiles were clustered using the mitotic network genes. Kaplan-Meier survival curves were generated for patients stratified into groups of high (upper tertile) and low (lower tertile) MNAI to evaluate differences in disease-free survival (DFS). For the cohort studied by Curtis et al. we similarly generated Kaplan-Meier survival curves to evaluate differences in disease-specific survival (DSS) and also fit a Cox proportional hazard model that included MNAI, age, size (spline function), lymph node positive (spline function), grade, stage, and PAM50 as variables. Statistical analyses were performed using the Statistical Package for the Social Sciences version 11.5 (SPSS, Inc., Chicago, IL, USA) and the R project for statistical computing (http://www.r-project.org/) with the packages “survival” and “rms”. Association analyses were performed based on one at a time analysis of variance (ANOVA) with copy number aberration (CNA) as the predictor variable for each mitotic net expression profile for both datasets from Chin et al*.* and Curtis et al. Differences in the MNAI index across various breast cancer subgroups was evaluated by ANOVA.

### Network construction and functional annotation

A network of genes found to be significantly correlated (Pearson correlation) with the mRNA expression levels of *PLK1, CENPE,* or *AURKB* was constructed based on the ExpressionCorrelation software tool (http://baderlab.org/Software/ExpressionCorrelation). Correlations exceeding a threshold were displayed as “edges” between two “nodes” (where nodes represent genes), and this approach was used to define the 54-gene mitotic apparatus network and assess it in datasets 1–4. Network figures were generated using Cytoscape version 2.5.1 (www.cytoscape.org). The gene ontology tool BiNGO [28] was employed to test for statistical enrichment of specific functional groups.

### Transcription factor binding site analysis

Ensembl and HGNC gene identifiers were obtained for the 54 mitotic network genes and their proximal promoter sequences were extracted using the ENSEMBL Biomart (GRCh37.p13). (http://grch37.ensembl.org/biomart/). Sequence regions 3000 immediately 5′ upstream from the transcription start site and 1000 Kb downstream (including the 5′ untranslated region (UTR)) were extracted. Transcription factor binding site (TFBS) matrices were obtained from the Transfac professional database (version 13.4) [29] and detected using the Match algorithm [30]. TFBS were detected with core and matrix similarity thresholds of 1 and 0.85 respectively, together with pre-calculated profiles that minimize both false positives and negatives.

In order to determine whether the transcription factors under investigation (MYC, ZEB1, FOXM1 and SOX9) bind to their predicted binding sites within mitotic network genes, we downloaded chromatin immunoprecipitation (ChIP)-seq datasets from the ENCODE project and other published studies spanning multiple human cell types from the NCBI Short Read Archive (SRA). As data from only one breast cancer cell line (MCF7) and non-transformed mammary cells (MCF10a) were available, we utilized high quality data from 22 datasets representing multiple human cell types immuno-precipitated with the four transcription factors under investigation. FASTQ files were aligned to a reference human genome (GRCh38) using the BWA algorithm and peaks were called using MACS2 to identify transcription factor binding sites. Bedtools (v2.25) software was used for intersect analysis with the putative promoter regions (-3 kb and +1 kb of TSS, GRCh38 annotation) of the 54 mitotic network genes.

### siRNA transfection and efficiency of knockdown

Small interfering RNAs (siRNAs) targeting mitotic genes (two siRNAs per gene), transcription factors and AllStars Negative Control siRNAs were purchased from Qiagen Inc. The AllStars Negative Control siRNA, which has no homology to any known mammalian genes, is the most thoroughly tested and validated negative control siRNA currently available. Breast cancer cells were seeded at the desired number in 96-well plates one day prior to transfection. Cells were transfected with 10 nM siRNAs using Dharmafect1 transfection regent (Dharmacon) according to the manufacturer’s instructions. After transfection with siRNAs for 72 hours, cell viability was measured using the CellTiter-Glo® assay (Promega). The RNA level of each gene and the actin control were measured with QuantiGene® 2.0 Reagent System (Panomics). The RNA levels relative to actin were compared to mRNA levels normalized to AllStars Negative control siRNA.

## Results

### Defining a mitotic apparatus transcriptional network

**Fig. 1:**
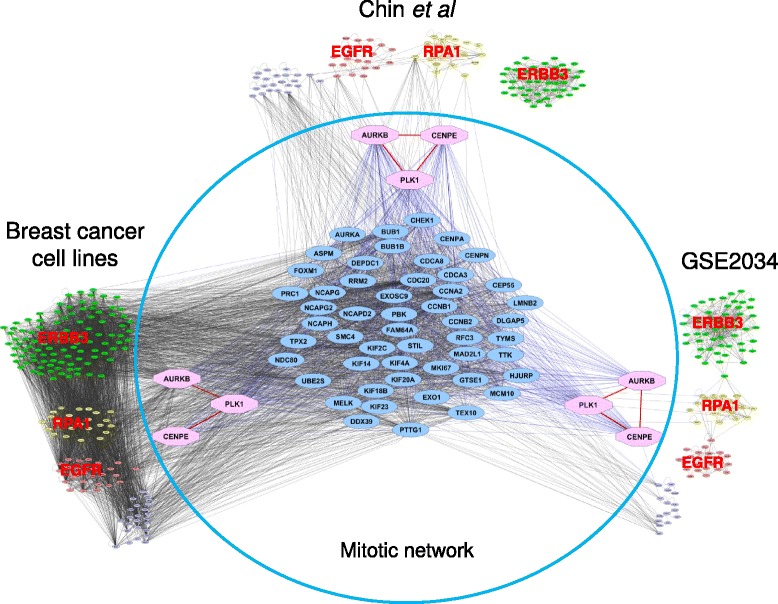
A conserved mitotic apparatus network in breast cancer cell lines and tumors. Transcripts with expression levels that correlated significantly with the expression levels of either *PLK1*, *CENPE*, or *AURKB* in 53 breast cancer cell lines (ArrayExpress (http://www.ebi.ac.uk/arrayexpress/) with accession number E-TABM-157) were identified. A relevance network was constructed based on the correlation between the resultant genes (272 Affymetrix probes), with edges drawn between significantly correlated genes (nodes). The mitotic gene network derived in the breast cancer cell lines was also confirmed in primary breast tumors from dataset 1 (as Chin et al) and dataset 2 (as GSE2034).

We analyzed gene expression profiles for 53 malignant and non-malignant human breast cancer cell lines [23] in order to identify the genes involved in mitotic apparatus function that had coordinately regulated transcriptional levels (i.e., increased or decreased together between samples). First, we searched for genes with expression levels that correlated significantly with the transcriptional levels of the mitotic apparatus genes *PLK1, CENPE,* and *AURKB* across the cell lines. This process defined a network comprising 272 Affymetrix probes representing 229 genes (*p* value of 2.5 × 10^−5^ based on 1000 permutation tests; Additional file 1: Table S1). Gene ontology analysis revealed that most of the genes in this network were involved in mitotic processes (Additional file 1: Table S2). We then assessed the extent to which this transcriptional network was coordinately regulated among samples from two separate primary breast tumor datasets [24, 31]. Figure 1 shows a mitotic apparatus network comprising 54 coordinately regulated transcripts in all three datasets (Additional file 1: Table S3). This mitotic apparatus network was also found to be co-regulated in cancers of the lung, ovary, prostate, brain, blood, and kidney (Additional file 2: Figure S1A-G), and in immortalized lymphoblast cell lines (Additional file 2: Figure S1H) and normal skin samples from crosses between *M. spretus* and *M. musculus* mouse strains [15].

**Fig. 2:**
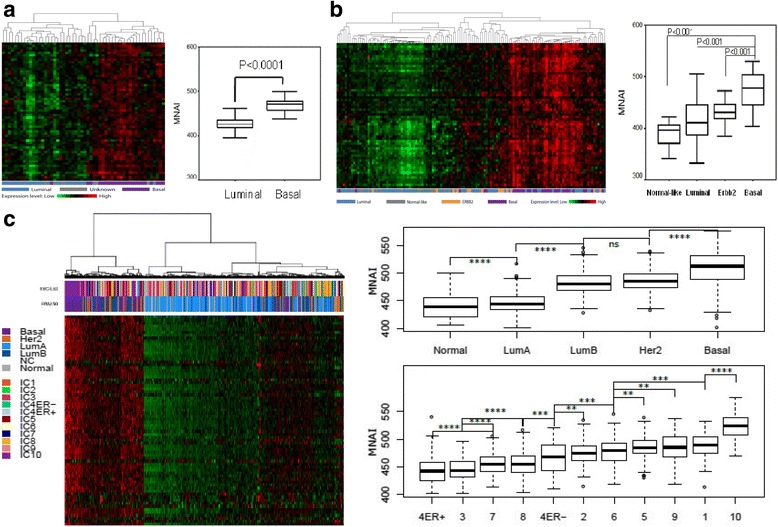
Mitotic network activity is elevated in a subset of breast cancers. Mitotic network activity index (*MNAI*) defined as the sum of the expression levels of the 54 mitotic network genes. *Heatmaps* illustrate mitotic network gene expression in breast cell lines (*n* = 53) [23] (basal vs. luminal subtype), *p* < 0.0001 (**a**), primary breast tumors from dataset 1 (*n* = 101) *p* < 0.001 (**b**), and primary tumors in the dataset from Curtis dataset. (*n* = 1980), *p* < 0.00001 (**c**), between basal and luminal tumors (PAM50) and integrated cluster 10 (*IC10*) vs. other subgroups (ICs) based on analysis of variance. *LumA* luminal A, *LumB* luminal B, *Her2* human epidermal growth factor receptor 2, *ER* estrogen receptor

**Fig. 3:**
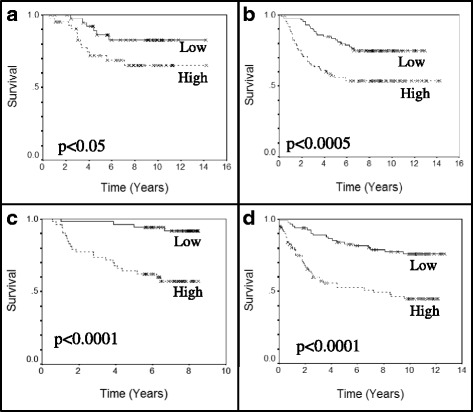
Association between mitotic network activity and survival time. *Kaplan-Meier curves* are shown for tumors with the highest one third of mitotic network activity index (MNAI) values and the lowest one third of MNAI values. Higher mitotic network activity was significantly associated with reduced survival time in four independent breast cancer studies based on the log-rank test. **a** Dataset 1, *p* < 0.05. **b** Dataset 2, *p* < 0.0005. **c** Dataset 3, *p* < 0.0001. **d** Dataset 4, *p* < 0.0001

We defined the mitotic network activity index (MNAI) as the sum of the transcriptional levels of the 54 coordinately regulated mitotic apparatus genes. Additional file 2: Figure S2 shows that the MNAI was significantly elevated in tumors relative to normal breast tissues despite considerable variability in the MNAI across tumors and in normal tissues. The MNAI was significantly higher in basal-like cell lines (Fig. 2a) and tumors (Figs. 2b-c) as compared to luminal subtype cell lines and tumors. In rank order the MNAI was lowest in normal-like tumors and luminal-A tumors, with progressively increasing MNAI values for luminal-B tumors, ERBB2-positive tumors and basal-like breast tumors (82 % of basal-like tumors exhibited high MNAI). High MNAI values were similarly enriched (95 % of cases) in integrative cluster 10 (IC10) (Fig. 2c). Figure 3 indicates that patients with high MNAI values had significantly shorter disease-free survival (DFS) than patients whose tumors had low MNAI values in four different breast cancer cohorts [24, 31–33], whereby patients were stratified by the upper and lower tertiles of MNAI values. Not surprisingly, the higher MNAI was significantly associated with the higher mitotic accounts defined as the number of mitotic events in 10 high power fields [34] (Additional file 2: Figure S3A) and shorter doubling time in cell lines (Additional file 2: Figure S3B-C). We further validated the association between MNAI and disease-specific survival (DSS) in the dataset of 1980 cases from Curtis et al., which remained significant after adjusting for standard clinical covariates, including molecular subtype (Additional file 2: Figure S4).

### Genomic mechanisms of mitotic apparatus network deregulation

**Fig. 4:**
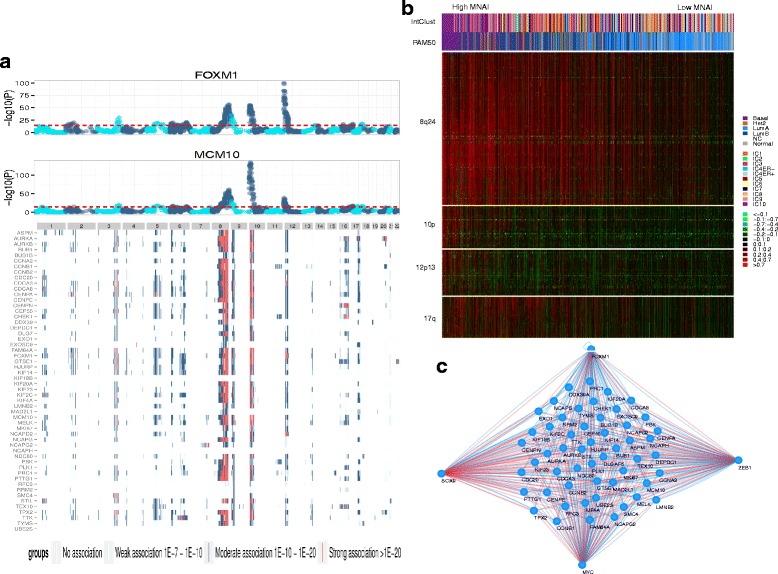
Genetic loci associated with mitotic network gene expression levels. **a** Genome-wide somatic copy number alterations associated with the expression of two mitotic network genes (FOXM1 and MCM10) are illustrated in a *Manhattan plot* (*upper panels*) above a *heatmap* indicating the strength of the association between mitotic network gene expression and genome-wide somatic copy number alterations in the dataset from Curtis et al. Each *row* in the *heatmap* represents a gene in the mitotic network and each *column* represents a chromosomal locus defined by merged copy number regions. *P* values indicating the significance of the association were based on analysis of variance for each gene, where *red* denotes genomic alterations strongly associated with the expression of mitotic network genes (*p* < 10^−20^), *blue* indicates moderate significance (10^−20^ < *p* < 10^−10^) and *green* shows significant, but slightly weaker association (10^−10^ < *p* < 10^−7^). **b** *Heatmap* representation of somatic copy number alterations for loci significantly associated with the expression of mitotic network genes. Amplified regions on chromosome 8q24 (120–132 Mb), 10p15-p12 (0–17.8 Mb), 12p13 (0–4 Mb), and 17q24-q25 (55.4–78.5 Mb) are indicated, where samples have been ordered by their mitotic network activity index. **c** These loci include the transcription factors MYC, ZEB1, FOXM1 and SOX9, each of which has predicted binding sites in multiple mitotic network genes, where edges connecting transcription factors to mitotic network genes based on binding site predictions are indicated in *red* and sites verified by ChIP-seq are shown in *blue. MNAI* mitotic network activity index

The genetically driven mitotic apparatus transcriptional network identified in mice [15] raised the possibility that genomic aberrations might contribute to increased mitotic activity in human cancer. We explored this possibility by searching for recurrent genome copy number abnormalities associated with elevated mitotic network gene expression in two separate breast cancer studies for which genomic and transcriptional profiles were available. In particular, we utilized data from a study by Chin et al.*,* which employed the Affymetrix U133 array to profile gene expression and a bacterial artificial chromosome (BAC) array to assay genomic copy number in 101 breast tumors and in a study by Curtis et al., which employed the Illumina HT12 Bead Array to profile gene expression and the Affymetrix SNP 6.0 platform to profile genome copy number in 1980 breast tumors. Strikingly, both studies showed that regions of amplification at chromosomes 8q24 (120–132 Mbp), 10p15-p12 (0–18 Mbp) and 12p13 (0–4 Mbp) were associated with increased expression of multiple genes in the mitotic apparatus network (Fig. 4, Additional file 2: Figure S5, Additional file 3: Table S5), attesting to the robustness of this signature. In addition, in the study by Curtis et al. there were strongly associated amplified regions on 17q24-q25 (55.4–78.5 Mbp). The strength of association between genomic copy number and expression in the dataset from Curtis et al. is shown in Fig. 4a for the *FOXM1* and *MCM10* genes (upper panel) and for all 54 mitotic genes in Additional file 4. The expression of *FOXM1* is associated with amplification of 12p13 where it maps, and with amplification of the other three regions of the genome. Genomic aberrations significantly associated with the expression levels of the 54 mitotic apparatus genes are indicated in Fig. 4a. All but two genes in the mitotic apparatus transcriptional network were associated with the same four regions of genome amplification in this dataset. The heatmap of copy number alterations shown in Fig. 4b reveals that the four regions encoding these genes are co-amplified in tumors with the highest MNAI. Intriguingly, these regions encode the transcription factors *MYC, ZEB1, SOX9,* and *FOXM1*, each of which has predicted binding sites in multiple genes comprising the mitotic apparatus network of 54 genes (Additional file 5: Table S6). Using publicly available ChIP-sequencing datasets from diverse cell lines (see “Methods”), we verified that MYC, FOXM1 and ZEB1 bind to all 54 mitotic network genes within the putative promoter region (−3 kb, +1 kb window around the transcriptional start site) and that ZEB1 binds to the putative promoters of 45 mitotic network genes.

### Therapeutic targeting of high MNAI tumors

**Fig. 5:**
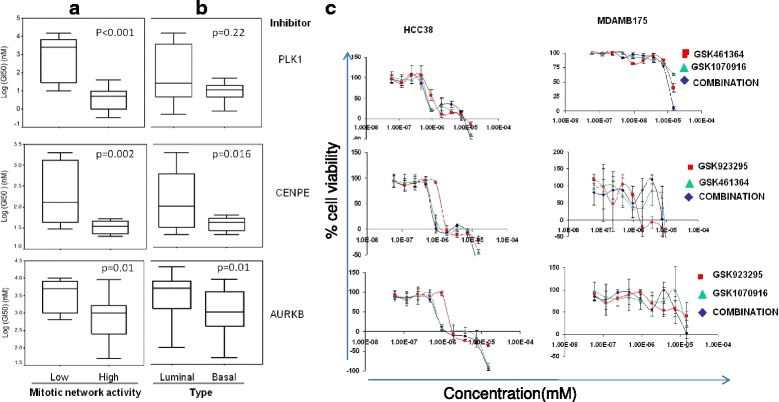
Dose response in breast cancer subtypes. Responses to GSK462364, GSK923295, and GSK1070916 were assayed for compounds individually or in combination in cell lines representing different breast cancer subtypes. **a** Responses in cell lines with a high mitotic network activity index (MNAI) and a low MNAI, and **b** responses in basal and luminal breast cancer cell lines were assessed using the two-tailed Mann-Whitney *U* test. **c** Responses to GSK462364, GSK923295 and GSK1070916 administered individually or in pairwise combination in breast cancer cell lines with high (HCC38) or low (MDAMB175) MNAI

We measured quantitative dose responses for 53 breast cancer cell lines to inhibitors of PLK1, CENPE and AURKB/C designated GSK462364 GSK923295 and GSK1070916, respectively (Additional file 6). The concentration required to inhibit growth by 50 % (GI_50_) after three days was used as the quantitative measure of response for each cell line. Importantly, the ensemble of cell lines mirrored many transcriptional and genomic features of primary breast tumors. The GI_50_ values varied widely among the cell lines (Additional file 2: Figure S7). Figure 5a shows that, on average, the GI_50_ values for GSK1070916, GSK462364, and GSK923295 were significantly lower in cell lines with a high MNAI as compared to cells with a low MNAI. This effect was slightly more pronounced than the difference between GI_50_ values for basal and luminal-like tumors (Fig. 5b).

Interestingly, the responses to the three compounds were significantly correlated among the cell lines (Additional file 1: Table S4). This suggests that drugs that target the mitotic apparatus may be clinically equivalent as they modulate the same biological function despite the fact that they inhibit different proteins involved in the function. As a result, combinations of mitotic apparatus inhibitors might not have additive or synergistic effects. We tested this by treating a sensitive (HCC38) and a resistant (MDAMB175) breast cancer cell line with GSK462364, GSK1070916, and GSK923295 alone and in pairwise combinations. As shown in Fig. 5c, the combination of compounds against two different mitotic apparatus proteins did not increase the response in either cell type. GSK462364, GSK923295, and GSK1070916 induced cell apoptosis in sensitive breast cancer cells in a dose-dependent manner assessed by immunofluorescence microscopy, but none of the combinations of two compounds significantly increased cell death (Additional file 2: Figure S8).

**Fig. 6:**
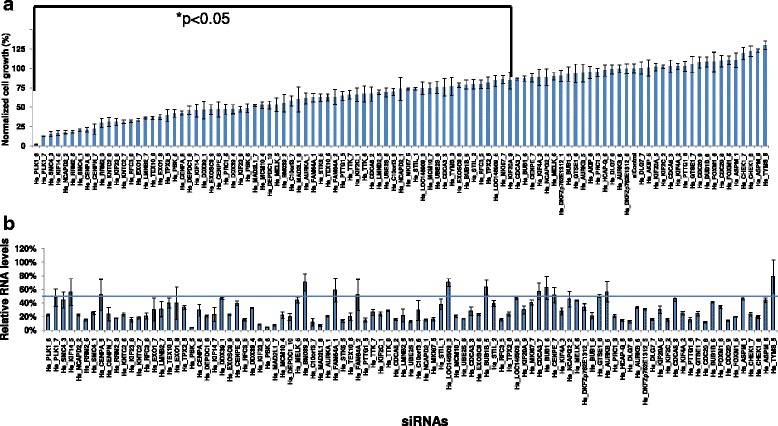
Small interfering RNA (*siRNA*) knockdown of mitotic network genes in the breast cancer cell line MDAMB231. Cells were transiently transfected with siRNAs targeting mitotic apparatus genes and cell viability, and mRNA levels were assayed. **a** Cell viability was measured after 72 hours and normalized to non-specific siRNA, which served as a negative control. siRNAs that induced significant growth inhibition (*p* < 0.05) relative to a control siRNA based on the two-tailed Student’s *t* test are indicated. **b** mRNA levels were quantified after siRNA knockdown by normalizing to mRNA levels after treatment with a control siRNA

In order to identify additional candidate therapeutic targets within the mitotic apparatus network, siRNAs were employed to knock down the expression of the 54 genes that comprise the network in MDAMB231 cells. MBAMD231 was chosen because of its high MNAI. There was more than 50 % knockdown of mRNA levels in 40 mitotic network genes (Fig. 6b). Figure 6a shows that siRNAs targeting 22 genes significantly diminished growth at 72 hours relative to that for a scrambled siRNA. The most inhibitory siRNAs targeted *PLK1*, the condensin complex component; *SMC4*, the kinesin family member; *KIF14*, the condensin complex regulatory subunit; *NCAPD2*, the condensing complex subunit 1 component; and the ribonucleotide reductase M2 subunit, *RRM2*. Protein motif analysis suggests that several of the 22 candidate therapeutic targets defined here are druggable (Additional file 1: Table S3) including the mitotic checkpoint protein kinase, *TTK;* the MAPKK-like protein kinase, *PBK* [35]; and *AURKA*, for which a small molecular inhibitor (MLN8054) [36] is already available and under evaluation in breast cancer cells.

## Discussion

Increased mitotic activity is a hallmark of aggressive cancer and is associated with genome instability, increased proliferative activity, and reduced patient survival in many types of cancer. In pursuit of mechanisms that increase mitotic activity in breast cancer, we identified a 54-gene mitotic apparatus network that is transcriptionally upregulated in primary tumors and breast cancer cell lines with high mitotic activity and/or high proliferative capacity. In parallel, we defined a mitotic network activity index (MNAI) as a quantitative measure of the transcriptional activity of the entire 54-gene network and showed that high MNAI is enriched amongst basal-like or IC10 tumors. We further showed that elevated MNAI is significantly associated with poor prognosis independent of standard clinical covariates.

Our data suggest that high MNAI and the elevated expression of the mitotic network genes can be explained, in part, by co-amplification of regions of chromosomes 8q24, 10p15-p12, 12p13, and 17q24-q25, which encode the transcription factors MYC, ZEB1, FOXM1, and SOX9, respectively. Indeed, each of the 54 genes in the mitotic apparatus network have predicted binding sites for one or more of these transcription factors and we verified the majority of binding sites for MYC, FOXM1, ZEB1, and SOX9 using publicly available ChIP-seq datasets. A genomic mechanism of mitotic apparatus network activation in cancer is consistent with the observation that the transcriptional activity of the mitotic network appears to be under genetic control in normal tissues both in the mouse [15] and human lymphocytes. These amplified transcription factors are known to play important roles in normal tissue development and/or stem cell biology. In particular, *MYC* has been implicated in reprogramming somatic cells to become pluripotent stem cells [37]. *ZEB1* has been associated with the epithelial-to-mesenchymal transition and cell migration [38]. *SOX9* has been implicated in neural crest tissue development, the maintenance of multipotency, and Notch-mediated cell fate determination [39, 40]. *FOXM1* is a transcription factor implicated in mitosis, a component of the 54-gene mitotic network, and a known transcriptional target of estrogen receptor alpha, with an important role in breast cancer endocrine biology [41]. These diverse functions may explain why tumors with high genome amplification-associated MNAI also have increased invasive potential and take on features associated with stem cells. Several cell proliferation signatures have been previously reported and shown to be associated with poor prognosis in subsets of breast cancer patients [42, 43], and not surprisingly these gene sets overlap with the MNAI. For example, many of the genes in the 54-gene mitotic apparatus network are included in the CIN25 gene signature reported to be associated with genome instability and reduced survival in multiple tumor types [4]. The association with genomic instability may stem from the deregulation of multiple mitotic apparatus genes via co-amplification of key transcription factors that influence genome instability both directly by interfering with DNA repair and the mechanical aspects of chromosome segregation and indirectly by deregulating checkpoint genes that normally function to inhibit cell cycle progression in cells with mechanical or genomic aberrations. Gene ontology analysis of the 54-gene mitotic apparatus signature indicates that *CCNB1, CENPE, DLGAP5, HJURP, KIF2C, NCAPD2, NCAPG, NCAPH, NDC80, PTTG1,* and *SMC4* are involved in mechanical aspects of chromosome segregation, whereas *CHEK1, EXO1, PTTG1, RFC3,* and *TYMS* are involved in DNA repair, and finally, *BUB1, BUB1B, CCNA2, CCNB1, CENPE, CHEK1, GSTE1, PLK1,* and *TTK* are cell cycle checkpoint genes.

Genome amplification-driven activation of the mitotic apparatus network raises the possibility that cancers with this mechanism of activation have become “addicted” to the activation and thus, will be more sensitive to agents that target the activated network proteins than tumors with lower activity. Consistent with this, we have shown that the small molecule inhibitors GSK462364, GSK923295, and GSK1070916 that target the network proteins PLK1, CENPE, and AURKB/C, respectively, inhibit the growth of breast cancer cell lines with high MNAI at lower concentrations than cell lines with low MNAI. These results also are supported by the report that treatment with the aurora kinase inhibitor, VX-680, selectively kills cells that over express MYC [44]. siRNA knockdown experiments show that inhibition of most genes in the mitotic apparatus network significantly represses growth, and implicate AURKA, TTK, MELK, and PBK as additional druggable proteins in the network. Based on our previous data (AACR 2008, Abstract# 2397) and others reported, these inhibitors not only induced the accumulation of cells with 4 *N* and ≥4 *N* DNA content, suggesting that DNA replication could occur in the absence of cytokinesis, indicative of a cell-cycle block in either G2 phase or mitosis, but also induced apoptosis in human cancer cell lines. Although GSK1070916 has potent activity against proliferating cells, a dramatic shift in potency is observed in primary, non-dividing tumors [45]. These observations indicate that mitotic apparatus inhibitors might be best targeted to aggressive cancers with high MNAI and/or co-amplification of *MYC, ZEB1, FOXM1,* and *SOX9*, thereby lowering the dose required for effective treatment and correspondingly lowering overall toxicity. Our studies of pairwise combinations of GSK462364, GSK923295, and GSK1070916 show that toxicity does not appear to be additive. Thus, combinations of compounds targeting multiple mitotic apparatus proteins might be deployed either together or sequentially to counter therapeutic resistance. This approach might lead to more durable treatment of the most aggressive forms of breast cancer.

## Conclusions

We presented evidence in this paper that high mitotic activity in a subset of breast cancers is caused, in part, by co-amplification of four regions of the genome that encode transcription factors that regulate a mitotic apparatus network playing important roles in cell cycle traverse, DNA repair and chromosome segregation. We defined a molecular signature that can be measured to identify tumors with high mitotic network activity and we showed that these tumors are likely to be preferentially sensitive to mitotic apparatus protein inhibitors, and combining mitotic apparatus protein inhibitors will reduce development of therapeutic resistance to these inhibitors.

## Additional files

Additional file 1: Tables S1, Tables S2, Tables S3 and Tables S4.
**Table S1.** List of genes that are significantly correlated with either PLK1, CENPE or AURKB in breast cancer cell lines and used for constructing gene network. **Table S2.** Gene ontology (GO) analysis of genes significantly correlated with PLK1, CENPE or AURKB in human breast cancer cell lines. **Table S3.** Mitotic network gene list. **Table S4.** Pearson coefficients for correlation (and significance) between cell line responses across 53 breast cell lines derived from tumor and normal tissues. The number of lines used to establish the correlation is listed below each correlation in parentheses. (DOC 282 kb) Additional file 2: Figures S1-S8.
**Figure S1.** The mitotic network is conserved across human malignancies including lung cancer [GEO:GSE3141] (Bild et al., 2006); ovarian cancer [GEO:GSE3149] (Bild et al., 2006); Wilms’ tumor [GEO:GSE10320] (Huang et al., 2009); prostate cancer [GEO:GSE8218]; glioblastomas [GEO:GSE13041] (Lee et al., 2008); acute lymphoblastic leukemia and acute myelogenous leukemia [GEO:GSE12417] (Metzeler et al., 2008); and lymphoblast cell lines [GEO:GSE11582] (Choy et al., 2008). **Figure S2.** Mitotic network activity is significantly higher in intraductal breast carcinoma (IDC) [GEO:GSE7307] as compared to normal tissues. **Figure S3.** **A** The activity of the mitotic network (MNAI) is significantly associated with pathological mitotic counts (*p* < 0.001) in the dataset from Chin et al. **B** Higher MNAI is also associated with shorter doubling time (*p* < 0.01). **C** The list of doubling time and MNAI value for each cell line. **Figure S4.** **A** Genomic alterations associated with the expression of mitotic network genes are summarized for the dataset from Chin et al. Each *row* represents a gene in the mitotic network and each *column* represents a chromosomal locus defined by a BAC array clone. *P* values indicating the significance of the association were based on ANOVA for each gene, where *red* denotes genetic alterations associated with the expression of mitotic network genes (*p* < 10-4), and *blue* indicates weaker association (10-4 < *p* < 10-3). **B** Common loci that are significantly associated with the expression of mitotic network genes are indicated for regions on chromosome 8 (23–33 Mb), 8 (115147 Mb), 10 (0–20 Mb), 12 (0–4 Mb). **Figure S5.** Illustration of putative transcription factor binding site motifs for MYC (*red*), SOX9 (*yellow*), FOXM1 (*blue*), and ZEB1 (*green*) within the core promoter region (–3000 and +1000) around the transcriptional start sites of the 54 mitotic network genes. **Figure S6.** **A** Association of mitotic network activity with disease-specific survival in the dataset from Curtis et al. Kaplan-Meier curves are shown for tumors stratified according to upper and lower tertiles of MNAI values. **B** Summary of a Cox proportional hazards model evaluating the association between MNAI and DSS, accounting for standard clinical covariates. **Figure S7.** The inhibitors of PLK, CENPE and AURKB/C selectively target a subset of basal-like human breast cancer cells. The GI_50_ values of GSK461364 (PLK1 inhibitor), GSK923295 (CENPE inhibitor), and GSK1070916 (Aurora kinase inhibitor) in breast cancer cells and nonmalignant mammary epithelial cells were ranked according to GI_50_values. **Figure S8.** The apoptotic effect was assessed using high-content imaging analysis. At 48 hours post treatment, cells were directly stained with 1 μmol/L YO-PRO-1 stain (Life Technologies) and 10 μg/mL Hoechst 33342 for 30 minutes at 37 °C. Apoptotic cells were detected and analyzed using multi-parameters of the Olympus Scan R microscope. The percentage of apoptosis was derived from the ratio of YO-PRO-1 positive cells to Hoechst 33342 staining for nuclei. **A** The representative images for each treatment; **B** the summery of % apoptosis. (PDF 2389 kb) Additional file 3: Table S5.
**Table S5**: Association of genomic aberrations and mitotic network gene expression in breast cancer. The p-values resulting from one-at-time ANOVA test for the association of each mitotic network genes with the 2995 copy number regions is indicated. See tsv file **Table S4**. (XLSX 2505 kb) Additional file 4: Figure S1. Manhattan plots for each mitotic network gene illustrating the strength of association (-log10 *p* value) between the expression of the gene and genome-wide somatic copy number alterations (see Fig S6). (PDF 8516 kb) Additional file 5: Table S6. List of ChIP-sequencing datasets evaluating MYC, FOXM1, ZEB1, and SOX9 binding in diverse cell lines and the corresponding GEO Accession IDs. (XLSX 9 kb) Additional file 6: Table S7. Cellular response of inhibitors in different doses after 72 hours of treatment. (XLSX 70 kb)
