# Erratum to: Genome co-amplification upregulates a mitotic gene network activity that predicts outcome and response to mitotic protein inhibitors in breast cancer

**DOI:** 10.1186/s13058-017-0809-6

**Published:** 2017-02-09

**Authors:** Zhi Hu, Jian-Hua Mao, Christina Curtis, Ge Huang, Shenda Gu, Laura Heiser, Marc E. Lenburg, James E. Korkola, Nora Bayani, Shamith Samarajiwa, Jose A. Seoane, Mark A. Dane, Amanda Esch, Heidi S. Feiler, Nicholas J. Wang, Mary Ann Hardwicke, Sylvie Laquerre, Jeff Jackson, Kenneth W. Wood, Barbara Weber, Paul T. Spellman, Samuel Aparicio, Richard Wooster, Carlos Caldas, Joe W. Gray

**Affiliations:** 1 Department of Biomedical Engineering, School of Medicine, Oregon Health & Science University, 3303 SW Bond Ave., CH13B, Portland, OR 97239 USA; 2 Life Sciences Division, Lawrence Berkeley National Laboratory, Berkeley, CA 94127 USA; 3 Department of Medicine, Division of Oncology and Department of Genetics, Stanford University School of Medicine, Stanford, CA 94305 USA; 4 Department of Pathology and Laboratory Medicine, Boston University School of Medicine, Boston, MA 02215 USA; 5 MRC Cancer Unit, University of Cambridge, Cambridge, CB2 0XZ UK; 6 GlaxoSmithKline, Collegeville, PA 19425 USA; 7 Cytokinetics, Inc., South San Francisco, CA 94080 USA; 8 Molecular Oncology, BC Cancer Research Centre, Vancouver, Canada; 9 Cancer Research UK, Cambridge Institute, Cambridge, UK

## Erratum

In the version of this article that was published on PubMed [1] the author’s name “Mark A. Dane” was formatted incorrectly in the XML mark up and therefore appeared incorrectly on PubMed. In the XML mark up, the middle initial “A” was added as a Particle when it should have been included as a Given Name. Due to this error, the author’s name was incorrectly formatted in PubMed as “A Dane M” and not as “Dane MA”. The author’s name is correct on the BioMed Central website. The author's name in the XML of the original article [1] has been updated accordingly.
