# Supplementary material for: Genome co-amplification upregulates a mitotic gene network activity that predicts outcome and response to mitotic protein inhibitors in breast cancer
Source: Breast Cancer Res. 2016 Jul 1;18:70. doi: 10.1186/s13058-016-0728-y (PMC4930593; doi:10.1186/s13058-016-0728-y)
Supplement: Additional file 1: Tables S1, Tables S2, Tables S3 and Tables S4. — Table S1. List of genes that are significantly correlated with either PLK1, CENPE or AURKB in breast cancer cell lines and used for constructing gene network. Table S2. Gene ontology (GO) analysis of genes significantly correlated with PLK1, CENPE or AURKB in human breast cancer cell lines. Table S3. Mitotic network gene list. Table S4. Pearson coefficients for correlation (and significance) between cell line responses across 53 breast cell lines derived from tumor and normal tissues. The number of lines used to establish the correlation is listed below each correlation in parentheses. (DOC 282 kb) [file 13058_2016_728_MOESM1_ESM.doc]

**Supplementary Table 1. List of genes that are significantly correlated with either PLK1, CENPE or AURKB in breast cancer cell lines and used for constructing gene network**

|  | Affy probe ID | Gene |
| --- | --- | --- |
| 1 | 200035_at | DULLARD |
| 2 | 200054_at | ZNF259 |
| 3 | 200600_at | MSN |
| 4 | 200634_at | PFN1 |
| 5 | 200670_at | XBP1 |
| 6 | 200804_at | TMBIM6 |
| 7 | 200815_s_at | PAFAH1B1 |
| 8 | 201215_at | PLS3 |
| 9 | 201272_at | AKR1B1 |
| 10 | 201276_at | RAB5B |
| 11 | 201427_s_at | SEPP1 |
| 12 | 201528_at | RPA1 |
| 13 | 201529_s_at | RPA1 |
| 14 | 201530_x_at | EIF4A1 |
| 15 | 201564_s_at | FSCN1 |
| 16 | 201584_s_at | DDX39 |
| 17 | 201663_s_at | SMC4 |
| 18 | 201697_s_at | DNMT1 |
| 19 | 201727_s_at | ELAVL1 |
| 20 | 201767_s_at | ELAC2 |
| 21 | 201770_at | SNRPA |
| 22 | 201774_s_at | NCAPD2 |
| 23 | 201844_s_at | RYBP |
| 24 | 201846_s_at | RYBP |
| 25 | 201983_s_at | EGFR |
| 26 | 202078_at | COPS3 |
| 27 | 202106_at | GOLGA3 |
| 28 | 202154_x_at | TUBB4 |
| 29 | 202159_at | FARSA |
| 30 | 202240_at | PLK1 |
| 31 | 202440_s_at | ST5 |
| 32 | 202454_s_at | ERBB3 |
| 33 | 202580_x_at | FOXM1 |
| 34 | 202589_at | TYMS |
| 35 | 202636_at | RNF103 |
| 36 | 202690_s_at | SNRPD1 |
| 37 | 202705_at | CCNB2 |
| 38 | 202734_at | TRIP10 |
| 39 | 202779_s_at | UBE2S |
| 40 | 202870_s_at | CDC20 |
| 41 | 202900_s_at | NUP88 |
| 42 | 203009_at | BCAM |
| 43 | 203065_s_at | CAV1 |
| 44 | 203306_s_at | SLC35A1 |
| 45 | 203317_at | PSD4 |
| 46 | 203324_s_at | CAV2 |
| 47 | 203362_s_at | MAD2L1 |
| 48 | 203418_at | CCNA2 |
| 49 | 203554_x_at | PTTG1 |
| 50 | 203701_s_at | TRMT1 |
| 51 | 203755_at | BUB1B |
| 52 | 203764_at | DLGAP5 |
| 53 | 203787_at | SSBP2 |
| 54 | 203871_at | SENP3 |
| 55 | 203895_at | PLCB4 |
| 56 | 203896_s_at | PLCB4 |
| 57 | 203906_at | IQSEC1 |
| 58 | 203961_at | NEBL |
| 59 | 203962_s_at | NEBL |
| 60 | 204088_at | P2RX4 |
| 61 | 204127_at | RFC3 |
| 62 | 204133_at | RRP9 |
| 63 | 204162_at | NDC80 |
| 64 | 204240_s_at | SMC2 |
| 65 | 204290_s_at | ALDH6A1 |
| 66 | 204317_at | GTSE1 |
| 67 | 204318_s_at | GTSE1 |
| 68 | 204420_at | FOSL1 |
| 69 | 204492_at | ARHGAP11A |
| 70 | 204567_s_at | ABCG1 |
| 71 | 204603_at | EXO1 |
| 72 | 204623_at | TFF3 |
| 73 | 204667_at | FOXA1 |
| 74 | 204709_s_at | KIF23 |
| 75 | 204822_at | TTK |
| 76 | 204825_at | MELK |
| 77 | 204887_s_at | PLK4 |
| 78 | 204942_s_at | ALDH3B2 |
| 79 | 204951_at | RHOH |
| 80 | 204962_s_at | CENPA |
| 81 | 204977_at | DDX10 |
| 82 | 205019_s_at | VIPR1 |
| 83 | 205046_at | CENPE |
| 84 | 205061_s_at | EXOSC9 |
| 85 | 205085_at | ORC1L |
| 86 | 205135_s_at | NUFIP1 |
| 87 | 205150_s_at | KIAA0644 |
| 88 | 205151_s_at | KIAA0644 |
| 89 | 205217_at | TIMM8A |
| 90 | 205248_at | DOPEY2 |
| 91 | 205251_at | PER2 |
| 92 | 205339_at | STIL |
| 93 | 205349_at | GNA15 |
| 94 | 205393_s_at | CHEK1 |
| 95 | 205394_at | CHEK1 |
| 96 | 205527_s_at | GEMIN4 |
| 97 | 205594_at | ZNF652 |
| 98 | 205652_s_at | TTLL1 |
| 99 | 205891_at | ADORA2B |
| 100 | 206034_at | SERPINB8 |
| 101 | 206364_at | KIF14 |
| 102 | 206445_s_at | PRMT1 |
| 103 | 206546_at | SYCP2 |
| 104 | 206571_s_at | MAP4K4 |
| 105 | 207030_s_at | CSRP2 |
| 106 | 207038_at | SLC16A6 |
| 107 | 207127_s_at | HNRNPH3 |
| 108 | 207949_s_at | ICA1 |
| 109 | 208079_s_at | AURKA |
| 110 | 208405_s_at | CD164 |
| 111 | 208456_s_at | RRAS2 |
| 112 | 208636_at | ACTN1 |
| 113 | 208637_x_at | ACTN1 |
| 114 | 208782_at | FSTL1 |
| 115 | 208789_at | PTRF |
| 116 | 208790_s_at | PTRF |
| 117 | 208827_at | PSMB6 |
| 118 | 208910_s_at | C1QBP |
| 119 | 208977_x_at | TUBB2A |
| 120 | 209110_s_at | RGL2 |
| 121 | 209161_at | PRPF4 |
| 122 | 209191_at | TUBB6 |
| 123 | 209195_s_at | ADCY6 |
| 124 | 209343_at | EFHD1 |
| 125 | 209350_s_at | GPS2 |
| 126 | 209408_at | KIF2C |
| 127 | 209464_at | AURKB |
| 128 | 209494_s_at | PATZ1 |
| 129 | 209642_at | BUB1 |
| 130 | 209747_at | TGFB3 |
| 131 | 209773_s_at | RRM2 |
| 132 | 209832_s_at | CDT1 |
| 133 | 210008_s_at | MRPS12 |
| 134 | 210024_s_at | UBE2E3 |
| 135 | 210052_s_at | TPX2 |
| 136 | 210108_at | CACNA1D |
| 137 | 210178_x_at | FUSIP1 |
| 138 | 210457_x_at | HMGA1 |
| 139 | 210463_x_at | TRMT1 |
| 140 | 210547_x_at | ICA1 |
| 141 | 210652_s_at | TTC39A |
| 142 | 210829_s_at | SSBP2 |
| 143 | 210916_s_at | CD44 |
| 144 | 210933_s_at | FSCN1 |
| 145 | 211034_s_at | C12orf51 |
| 146 | 211084_x_at | PRKD3 |
| 147 | 211126_s_at | CSRP2 |
| 148 | 211160_x_at | ACTN1 |
| 149 | 211519_s_at | KIF2C |
| 150 | 211750_x_at | TUBA1A |
| 151 | 211787_s_at | EIF4A1 |
| 152 | 211954_s_at | IPO5 |
| 153 | 211964_at | COL4A2 |
| 154 | 211982_x_at | XPO6 |
| 155 | 212021_s_at | MKI67 |
| 156 | 212097_at | CAV1 |
| 157 | 212099_at | RHOB |
| 158 | 212148_at | PBX1 |
| 159 | 212151_at | PBX1 |
| 160 | 212181_s_at | NUDT4 |
| 161 | 212183_at | NUDT4 |
| 162 | 212190_at | SERPINE2 |
| 163 | 212378_at | GART |
| 164 | 212441_at | KIAA0232 |
| 165 | 212442_s_at | LASS6 |
| 166 | 212446_s_at | LASS6 |
| 167 | 212450_at | SECISBP2L |
| 168 | 212508_at | MOAP1 |
| 169 | 212590_at | RRAS2 |
| 170 | 212789_at | NCAPD3 |
| 171 | 212841_s_at | PPFIBP2 |
| 172 | 212856_at | DIP |
| 173 | 212949_at | NCAPH |
| 174 | 212956_at | TBC1D9 |
| 175 | 213172_at | TTC9 |
| 176 | 213198_at | ACVR1B |
| 177 | 213226_at | EXOSC9 |
| 178 | 213302_at | PFAS |
| 179 | 213308_at | SHANK2 |
| 180 | 213412_at | TJP3 |
| 181 | 213441_x_at | SPDEF |
| 182 | 213476_x_at | TUBB4 |
| 183 | 213651_at | INPP5J |
| 184 | 213784_at | RABL4 |
| 185 | 214214_s_at | C1QBP |
| 186 | 214266_s_at | PDLIM7 |
| 187 | 214404_x_at | SPDEF |
| 188 | 214433_s_at | SELENBP1 |
| 189 | 214700_x_at | RIF1 |
| 190 | 214710_s_at | CCNB1 |
| 191 | 214746_s_at | ZNF467 |
| 192 | 214784_x_at | XPO6 |
| 193 | 215113_s_at | SENP3 |
| 194 | 215942_s_at | GTSE1 |
| 195 | 216602_s_at | FARSA |
| 196 | 216952_s_at | LMNB2 |
| 197 | 217099_s_at | GEMIN4 |
| 198 | 217368_at | RP11-385M4.4 |
| 199 | 217640_x_at | C18orf24 |
| 200 | 217943_s_at | MAP7D1 |
| 201 | 217979_at | TSPAN13 |
| 202 | 217992_s_at | EFHD2 |
| 203 | 217996_at | PHLDA1 |
| 204 | 218009_s_at | PRC1 |
| 205 | 218035_s_at | RBM47 |
| 206 | 218104_at | TEX10 |
| 207 | 218156_s_at | TSR1 |
| 208 | 218204_s_at | FYCO1 |
| 209 | 218355_at | KIF4A |
| 210 | 218502_s_at | TRPS1 |
| 211 | 218512_at | WDR12 |
| 212 | 218542_at | CEP55 |
| 213 | 218574_s_at | LMCD1 |
| 214 | 218584_at | TCTN1 |
| 215 | 218662_s_at | NCAPG |
| 216 | 218663_at | NCAPG |
| 217 | 218710_at | TTC27 |
| 218 | 218726_at | HJURP |
| 219 | 218755_at | KIF20A |
| 220 | 218770_s_at | TMEM39B |
| 221 | 218828_at | PLSCR3 |
| 222 | 218854_at | SART2 |
| 223 | 218918_at | MAN1C1 |
| 224 | 219098_at | MYBBP1A |
| 225 | 219148_at | PBK |
| 226 | 219204_s_at | SRR |
| 227 | 219206_x_at | TMBIM4 |
| 228 | 219223_at | C9orf7 |
| 229 | 219555_s_at | CENPN |
| 230 | 219562_at | RAB26 |
| 231 | 219570_at | KIF16B |
| 232 | 219588_s_at | NCAPG2 |
| 233 | 219918_s_at | ASPM |
| 234 | 219956_at | GALNT6 |
| 235 | 220173_at | C14orf45 |
| 236 | 220192_x_at | SPDEF |
| 237 | 220258_s_at | WDR79 |
| 238 | 220295_x_at | DEPDC1 |
| 239 | 220306_at | FAM46C |
| 240 | 220651_s_at | MCM10 |
| 241 | 220658_s_at | ARNTL2 |
| 242 | 221024_s_at | SLC2A10 |
| 243 | 221260_s_at | CSRNP2 |
| 244 | 221436_s_at | CDCA3 |
| 245 | 221510_s_at | GLS |
| 246 | 221520_s_at | CDCA8 |
| 247 | 221561_at | SOAT1 |
| 248 | 221588_x_at | ALDH6A1 |
| 249 | 221589_s_at | ALDH6A1 |
| 250 | 221591_s_at | FAM64A |
| 251 | 221598_s_at | MED27 |
| 252 | 221655_x_at | EPS8L1 |
| 253 | 221676_s_at | CORO1C |
| 254 | 221832_s_at | LUZP1 |
| 255 | 221845_s_at | CLPB |
| 256 | 221849_s_at | LOC90379 |
| 257 | 221880_s_at | FAM174B |
| 258 | 221934_s_at | DALRD3 |
| 259 | 221987_s_at | TSR1 |
| 260 | 222039_at | KIF18B |
| 261 | 222125_s_at | P4HTM |
| 262 | 35148_at | TJP3 |
| 263 | 35666_at | SEMA3F |
| 264 | 40093_at | BCAM |
| 265 | 44563_at | WRAP53 |
| 266 | 50376_at | ZNF444 |
| 267 | 50965_at | RAB26 |
| 268 | 51158_at | FAM174B |
| 269 | 51176_at | MED27 |
| 270 | 56197_at | PLSCR3 |
| 271 | 61874_at | C9orf7 |
| 272 | 91826_at | EPS8R1 |

**Supplementary Table 2. Gene Ontology (GO) analysis for genes** significantly correlated with PLK1, CENPE or AURKB in human breast cancer cell lines

| GO ID | Description | Gene |
| --- | --- | --- |
| 7067 | mitosis | KIF23 TUBB2A AURKA CEP55 AURKB PTTG1 KIF2C CDCA8 NCAPH C18ORF24 NCAPG2 NCAPG BUB1 PAFAH1B1 FOSL1 CCNA2 ASPM CDCA3 TUBB4 DLGAP5 TPX2 CENPE CDC20 NDC80 PLK PBK SMC2 NCAPD3 SMC4 NCAPD2 CCNB1 MAD2L1 CCNB2 BUB1B |
| 279 | M phase | KIF23 TUBB2A CHEK1 AURKA CEP55 AURKB PTTG1 SYCP2 RPA1 KIF2C CDCA8 NCAPH C18ORF24 NCAPG2 NCAPG BUB1 PAFAH1B1 FOSL1 CCNA2 ASPM CDCA3 TUBB4 EXO1 DLGAP5 TPX2 CENPE NDC80 CDC20 PLK PBK SMC2 NCAPD3 SMC4 NCAPD2 CCNB1 MAD2L1 CCNB2 BUB1B |
| 87 | M phase of mitotic cell cycle | KIF23 TUBB2A AURKA CEP55 AURKB PTTG1 KIF2C CDCA8 NCAPH C18ORF24 NCAPG2 NCAPG BUB1 PAFAH1B1 FOSL1 CCNA2 ASPM CDCA3 TUBB4 DLGAP5 TPX2 CENPE CDC20 NDC80 PLK PBK SMC2 NCAPD3 SMC4 NCAPD2 CCNB1 MAD2L1 CCNB2 BUB1B |
| 278 | mitotic cell cycle | KIF23 PRC1 TUBB2A TTK CHEK1 AURKA CEP55 AURKB PTTG1 GTSE1 ACVR1B KIF2C CDCA8 NCAPH C18ORF24 PSMB6 NCAPG2 NCAPG BUB1 PAFAH1B1 FOSL1 CCNA2 ASPM CDCA3 TUBB4 DLGAP5 TPX2 CENPE NDC80 CDC20 PLK PBK SMC2 NCAPD3 SMC4 NCAPD2 CCNB1 MAD2L1 CCNB2 BUB1B |
| 22403 | cell cycle phase | KIF23 TUBB2A CHEK1 AURKA CEP55 AURKB PTTG1 SYCP2 GTSE1 RPA1 ACVR1B KIF2C CDCA8 NCAPH C18ORF24 NCAPG2 NCAPG BUB1 PAFAH1B1 FOSL1 CCNA2 ASPM CDCA3 TUBB4 EXO1 DLGAP5 TPX2 CENPE NDC80 CDC20 PLK PBK SMC2 NCAPD3 SMC4 NCAPD2 CCNB1 MAD2L1 CCNB2 BUB1B |
| 22402 | cell cycle process | KIF23 PRC1 TUBB2A TTK CHEK1 AURKA CEP55 AURKB PTTG1 SYCP2 GTSE1 RPA1 ACVR1B KIF2C CDCA8 NCAPH C18ORF24 PSMB6 NCAPG2 NCAPG BUB1 PAFAH1B1 FOSL1 CCNA2 ASPM CDCA3 TUBB4 EXO1 DLGAP5 TPX2 CENPE NDC80 CDC20 PLK PBK SMC2 NCAPD3 SMC4 NCAPD2 CCNB1 MAD2L1 CCNB2 BUB1B |
| 7049 | cell cycle | KIF23 PRC1 TUBB2A TTK CHEK1 AURKA CEP55 AURKB PTTG1 SYCP2 GTSE1 CDT1 RPA1 ACVR1B KIF2C CDCA8 NCAPH C18ORF24 PSMB6 NCAPG2 NCAPG BUB1 PAFAH1B1 FOSL1 CCNA2 ASPM CDCA3 TUBB4 EXO1 MKI67 DLGAP5 TPX2 CENPE NDC80 CDC20 PLK PBK SMC2 NCAPD3 GPS2 SMC4 NCAPD2 CCNB1 CCNB2 MAD2L1 RIF1 BUB1B |
| 51301 | cell division | KIF23 PRC1 AURKB PTTG1 CEP55 SYCP2 NCAPH CDCA8 C18ORF24 NCAPG NCAPG2 BUB1 PAFAH1B1 CCNA2 ASPM CDCA3 CENPE CDC20 NDC80 PLK SMC2 NCAPD3 SMC4 NCAPD2 CCNB1 MAD2L1 CCNB2 BUB1B |
| 6996 | organelle organization and biogenesis | KIF23 CAV2 CAV1 KIF4A RAB5B PRC1 TUBB2A TTK AURKA PTTG1 SYCP2 GTSE1 KIF2C PFN1 MOAP1 NCAPH CENPA NCAPG2 NCAPG DULLARD TUBB6 PAFAH1B1 DOPEY2 TUBA1A TRIP10 GEMIN4 PLS3 TUBB4 KIF14 EXOSC9 TSR1 DLGAP5 FSCN1 KIF18B ACTN1 CENPE NDC80 RRP9 C20ORF23 DIP SMC2 HMGA1 NCAPD3 SMC4 TIMM8A NCAPD2 BUB1B KIF20A |
| 16359 | mitotic chromosome segregation | NCAPH NCAPG NCAPG2 DLGAP5 CENPE NDC80 SMC2 NCAPD3 SMC4 NCAPD2 |
| 819 | sister chromatid segregation | NCAPH NCAPG NCAPG2 DLGAP5 CENPE NDC80 SMC2 NCAPD3 SMC4 NCAPD2 |
| 7017 | microtubule-based process | KIF14 KIF23 KIF4A PRC1 TUBB2A KIF18B TTK CENPE AURKA NDC80 C20ORF23 GTSE1 KIF2C BUB1B TUBB6 PAFAH1B1 TUBA1A TUBB4 KIF20A |
| 16043 | cell organization and biogenesis | KIF23 PDLIM7 RAB5B PRC1 TUBB2A SNRPD1 TTK AURKA PTTG1 GTSE1 EFHD1 KIF2C TUBB6 TUBA1A PLS3 TUBB4 EGFR KIF14 EXOSC9 ACTN1 RRP9 C20ORF23 HMGA1 NCAPD3 NCAPD2 TIMM8A BUB1B FUSIP1 CAV2 KIF4A CAV1 SYCP2 PFN1 NCAPH MOAP1 NCAPG NCAPG2 CENPA DULLARD PAFAH1B1 DOPEY2 TRIP10 GEMIN4 COL4A2 TSR1 DLGAP5 FSCN1 KIF18B CENPE NDC80 DIP SMC2 SMC4 CORO1C PLSCR3 KIF20A |
| 7059 | chromosome | NCAPH NCAPG NCAPG2 DLGAP5 CENPE NDC80 PTTG1 SMC2 NCAPD3 SMC4 NCAPD2 |

**Supplementary Table 3. Mitotic network gene list**

| Gene Symbol | Gene full name | Accession number | Reference | Druggable? |
| --- | --- | --- | --- | --- |
| AURKA | Homo sapiens aurora kinase A,transcript variant 2 | NM_003600 | Macůrek L 2008 | Y |
| AURKB | Homo sapiens aurora kinase B (AURKB) | NM_004217 | Shannon KB 2002 | Y |
| BUB1 | Homo sapiens BUB1 budding uninhibited by benzimidazoles 1 homolog (yeast) | NM_004336 | Kang J 2008 | Y |
| CENPE | Homo sapiens centromere protein E | NM_001813 | Yardimci H 2008; | Y |
| CHEK1 | Homo sapiens CHK1 checkpoint homolog (S. pombe) | NM_001274 | Rodriguez R 2005 | Y |
| FOXM1 | Homo sapiens forkhead box M1 (FOXM1), transcript variant 1 | NM_202002 | Wonsey DR 2005; Fu Z 2008 | Y |
| MELK | Homo sapiens maternal embryonic leucine zipper kinase | NM_014791 | Badouel C 2006 | Y |
| PBK | MAPKK-like protein kinase; PDZ-binding kinase; T-LAK cell-originated protein kinase; | NM_018492 | Gaudet S 2000; Simons-Evelyn M 2001 | Y |
| PLK1 | Homo sapiens polo-like kinase 1 | NM_005030 | Archambault V 2009; Kishi K 2009; Fu Z 2008 | Y |
| TTK | Homo sapiens TTK protein kinase | NM_003318 | Liu ST 2003 | Y |
| TYMS | Homo sapiens thymidylate synthetase | NM_001071 | Kemming D 2006; Le X 2004 | Y |
| ASPM | Homo sapiens asp (abnormal spindle) homolog, microcephaly associated (Drosophila) | NM_018136 | Bond J 2002 |  |
| BUB1B | Homo sapiens BUB1 budding uninhibited by benzimidazoles 1 homolog beta (yeast) | NM_001211 | Lampson MA 2004 |  |
| CCNA2 | Homo sapiens cyclin A2 | NM_001237 | Wolthuis R 2008 |  |
| CCNB1 | Homo sapiens cyclin B1 | NM_031966 | Allan LA 2007 |  |
| CCNB2 | Homo sapiens cyclin B2 | NM_004701 | Bellanger S 2007 |  |
| CDC20 | Homo sapiens cell division cycle 20 homolog (S. cerevisiae) | NM_001255 | Liu H 2007 |  |
| CDCA3 | cell division cycle associated 3(also named TOME-1) | NM_031299 | Ayad NG 2003 |  |
| CDCA8 | Homo sapiens cell division cycle associated 8 | NM_018101 | Slattery SD 2008 |  |
| CENPA | centromere protein A, isoform a | NM_001809 | Black BE 2007, McClelland SE 2007 |  |
| CENPN | centromere protein N | NM_001100625 | Foltz DR 2006 |  |
| CEP55 | centrosomal protein 55kDa | NM_018131 | Zhao WM 2006; Morita E 2007 |  |
| DDX39 | DEAD (Asp-Glu-Ala-Asp) box polypeptide 39 | NM_005804 |  |  |
| DEPDC1 | DEP domain containing 1 | NM_017779 |  |  |
| DLGAP5 | Homo sapiens discs, large (Drosophila) homolog-associated protein 5 | NM_014750 |  |  |
| EXO1 | exonuclease 1 | NM_006027.3 | Fiorentini P 1997 |  |
| EXOSC9 | Homo sapiens exosome component 9 (EXOSC9) | NM_001034194 |  |  |
| FAM64A | family with sequence similarity 64, member A | NM_019013 |  |  |
| GTSE1 | Homo sapiens G-2 and S-phase expressed 1 | NM_016426 | Monte M 2003 |  |
| HJURP | Homo sapiens Holliday junction recognition protein | NM_018410 | Kato T 2007 |  |
| KIF14 | kinesin family member 14 | NM_014875 | Carleton M 2006; Corson TW 2007 |  |
| KIF18B | Homo sapiens kinesin family member 18B | NM_001080443 | Miki H 2005 |  |
| KIF20A | Homo sapiens kinesin family member 20A | NM_005733 | Neef R 2003 |  |
| KIF23 | Homo sapiens kinesin family member 23 (KIF23), transcript variant 1 | NM_138555 | Lee KS 1995 |  |
| KIF2C | Homo sapiens kinesin family member 2C | NM_006845 | Manning AL 2007 |  |
| KIF4A | Homo sapiens kinesin family member 4A | NM_012310 | Mazumdar M 2004 |  |
| LMNB2 | lamin B2 | NM_032737 | Tsai MY, 2006 |  |
| MAD2L1 | MAD2 mitotic arrest deficient-like 1 | NM_002358 | Tighe A, 2008; Lee SH, 2008 |  |
| MCM10 | minichromosome maintenance complex component 10 | NM_182751 | Park JH, 2008 |  |
| MKI67 | antigen identified by monoclonal antibody Ki-67(Ki-67) | NM_002417 | Schluter C 1993 |  |
| NCAPD2 | non-SMC condensin I complex, subunit D2 | NM_014865 | Ball AR Jr, 2002 |  |
| NCAPG | Homo sapiens non-SMC condensin I complex, subunit G | NM_022346 | Murphy LA 2008 |  |
| NCAPG | non-SMC condensin I complex, subunit G | NM_022346. | Murphy LA, 2008 |  |
| NCAPG2 | Homo sapiens non-SMC condensin II complex, subunit G2 | NM_017760 |  |  |
| NCAPH | Homo sapiens non-SMC condensin I complex, subunit H | NM_015341 |  |  |
| NDC80 | NDC80 homolog, kinetochore complex component (S. cerevisiae) | NM_006101 | McCleland ML 2003, Wei RR 2007 |  |
| PRC1 | Homo sapiens protein regulator of cytokinesis 1 (PRC1) | NM_199414 | Jiang W, 1998 |  |
| PTTG1 | Homo sapiens pituitary tumor-transforming 1 | NM_004219 | Zou 1999; Ying H 2006 |  |
| RFC3 | replication factor C (activator 1) 3, 38kDa | NM_181558 | Shimada M 1999 |  |
| RRM2 | ribonucleotide reductase M2 polypeptide | NM_001034.2 | PMID: 12615712 |  |
| SMC4 | structural maintenance of chromosomes 4 | NM_001002800 | Hagstrom KA 2002; Steffensen S 2001 |  |
| STIL | Homo sapiens SCL/TAL1 interrupting locus (STIL) | NM_001048166 | Erez A, 2004; Campaner S 2005 |  |
| TEX10 | testis expressed 10 | NM_017746 |  |  |
| TPX2 | Homo sapiens TPX2, microtubule-associated, homolog (Xenopus laevis) | NM_012112 | Bayliss R 2003 |  |
| UBE2S | ubiquitin-conjugating enzyme E2S | NM_014501 | Dephoure N 2008 |  |

**Supplemental Table 4:** **Association of genomic aberrations and mitotic network gene expression in breast cancer.** The p-values resulting from one-at-time ANOVA test for the association of each mitotic network genes with the 2995 copy number regions is indicated. See tsv file Table S4.

See excel file Table S4

**Supplemental Table 5. List of ChIP-sequencing datasets** evaluating MYC, FOXM1, ZEB1, and SOX9 binding in diverse cell lines and the corresponding GEO Accession IDs.

See excel file Table S5

**Supplemental Table 6. Cellular response of inhibitors in different doses after 72 hour treatment**

See excel file Table S6

**Supplemental Table 7. Pearson correlation coefficients (and significance) between cell line responses across 53 breast cell lines derived from tumor and normal tissues**. The number of lines used to establish the correlation is listed below each correlation in parentheses.

|  | GSK1070916 | GSK461364 | GSK923295 |
| --- | --- | --- | --- |
| GSK1070916 | 1.0 | **0.326* (0.024^#^)**  (N=46) | **0.323 (0.025)**  (N=47) |
| GSK461364 |  | 1.0 | **0.444 (0.01)**  (N=50) |
| GSK923295 |  |  | 1.0 |

* Pearson correlation coefficient; ^#^ p-value
