# Supplementary material for: Genome co-amplification upregulates a mitotic gene network activity that predicts outcome and response to mitotic protein inhibitors in breast cancer
Source: Breast Cancer Res. 2016 Jul 1;18:70. doi: 10.1186/s13058-016-0728-y (PMC4930593; doi:10.1186/s13058-016-0728-y)
Supplement: Additional file 2: Figures S1-S8. — Figure S1. The mitotic network is conserved across human malignancies including lung cancer [GEO:GSE3141] (Bild et al., 2006); ovarian cancer [GEO:GSE3149] (Bild et al., 2006); Wilms’ tumor [GEO:GSE10320] (Huang et al., 2009); prostate cancer [GEO:GSE8218]; glioblastomas [GEO:GSE13041] (Lee et al., 2008); acute lymphoblastic leukemia and acute myelogenous leukemia [GEO:GSE12417] (Metzeler et al., 2008); and lymphoblast cell lines [GEO:GSE11582] (Choy et al., 2008). Figure S2. Mitotic network activity is significantly higher in intraductal breast carcinoma (IDC) [GEO:GSE7307] as compared to normal tissues. Figure S3. A The activity of the mitotic network (MNAI) is significantly associated with pathological mitotic counts (p < 0.001) in the dataset from Chin et al. B Higher MNAI is also associated with shorter doubling time (p < 0.01). C The list of doubling time and MNAI value for each cell line. Figure S4. A Genomic alterations associated with the expression of mitotic network genes are summarized for the dataset from Chin et al. Each row represents a gene in the mitotic network and each column represents a chromosomal locus defined by a BAC array clone. P values indicating the significance of the association were based on ANOVA for each gene, where red denotes genetic alterations associated with the expression of mitotic network genes (p < 10-4), and blue indicates weaker association (10-4 < p < 10-3). B Common loci that are significantly associated with the expression of mitotic network genes are indicated for regions on chromosome 8 (23–33 Mb), 8 (115147 Mb), 10 (0–20 Mb), 12 (0–4 Mb). Figure S5. Illustration of putative transcription factor binding site motifs for MYC (red), SOX9 (yellow), FOXM1 (blue), and ZEB1 (green) within the core promoter region (–3000 and +1000) around the transcriptional start sites of the 54 mitotic network genes. Figure S6. A Association of mitotic network activity with disease-specific survival in the dataset from Curtis et al. Kaplan [file 13058_2016_728_MOESM2_ESM.pdf]

Supplemental Figure 1. The mitotic network is conserved across human malignancies including lung cancer, GEO accession number GSE3141 (Bild et al., 2006); ovarian cancer, GEO accession number GSE3149 (Bild et al., 2006); Wilms' tumor, GEO accession number GSE10320 (Huang et al., 2009); prostate cancer, GEO accession number GSE8218; glioblastomas, GEO accession number GSE13041 (Lee et al., 2008); acute lymphoblastic leukemia and acute myelogenous leukemia, GEO accession number, GSE12417 (Metzeler et al., 2008); and lymphoblast cell lines, GEO accession number, GSE11582 (Choy et al., 2008).

Supplemental Figure 2. Mitotic network activity is significantly higher in intraductal breast carcinoma (IDC) (GEO accession number, GSE7307) as compared to normal tissues.

Supplemental Figure 3. (A) The activity of the mitotic network (MNAI) is significantly associated with pathological mitotic counts, $p<0.001$ in the Chin *et al* dataset. (B) Higher MNAI is also associated with shorter doubling time, $p<0.01$.(C) The list of doubling time and MNAI value for each cell line.

Supplemental Figure 4. (A) Genomic alterations associated with the expression of mitotic network genes are summarized for the Chin *et al* dataset. Each row represents a gene in the mitotic network and each column represents a chromosomal locus defined by a BAC array clone. P-values indicating the significance of the association were based on ANOVA tests for each gene, where red denotes genetic alterations associated with the expression of mitotic network genes ($p < 10^{-4}$), and blue indicates weaker associations ($10^{-4}<p<10^{-3}$). (B) Common loci that are significantly associated with the expression of mitotic network genes are indicated for regions on chromosome 8 (23-33Mb), 8 (115-147Mb), 10 (0-20Mb), 12 (0-4Mb).

Supplemental Figure 5. Illustration of putative transcription factor binding site motifs for MYC (red), SOX9 (yellow), FOXM1 (blue), and ZEB1 (green) within the core promoter region (-3000 and +1000) around the transcriptional start sites of the 54 mitotic network genes.

Supplemental Figure 6. Manhattan plots for each mitotic network gene illustrating the strength of association (-log10 p-value) between the expression of the gene and genome-wide somatic copy number alterations.(see Fig S6)

Supplemental Figure 7. (A) Association of mitotic network activity with disease-specific survival in the Curtis *et al*. dataset. Kaplan-Meier curves are shown for tumors stratified according to upper and lower tertiles of MNAI values. (B) Summary of Cox proportional hazards model evaluating the association between MNAI and DSS accounting for standard clinical covariates.

Supplemental Figure 8. The inhibitors of PLK, CENPE and AURKB/C selectively target a subset of basal-like human breast cancer cells. The GI_{50} Values of GSK461364 (PLK1 inhibitor), GSK923295 (CENPE inhibitor) and GSK1070916 (Aurora kinase inhibitor) in breast cancer cells and non-malignant mammary epithelial cells were ranked according to GI_{50} values.

Supplemental Figure 9. The apoptotic effect was assessed using high-content imaging analysis. After 48 hours post treatment, cells were directly stained with 1 µmol/L YO-PRO-1 stain (Life Technologies) and 10 µg/mL Hoechst 33342 for 30 min at 37°C. Apoptotic cells were detected and analyzed using multi-parameters of Olympus ScanR microscope. The percentage of apoptosis was derived from the ratio of YO-PRO-1 positive cells to Hoechst 33342 staining for nuclei. (**A)** the representative images for each treatment; (**B**) the summery of % apoptosis.

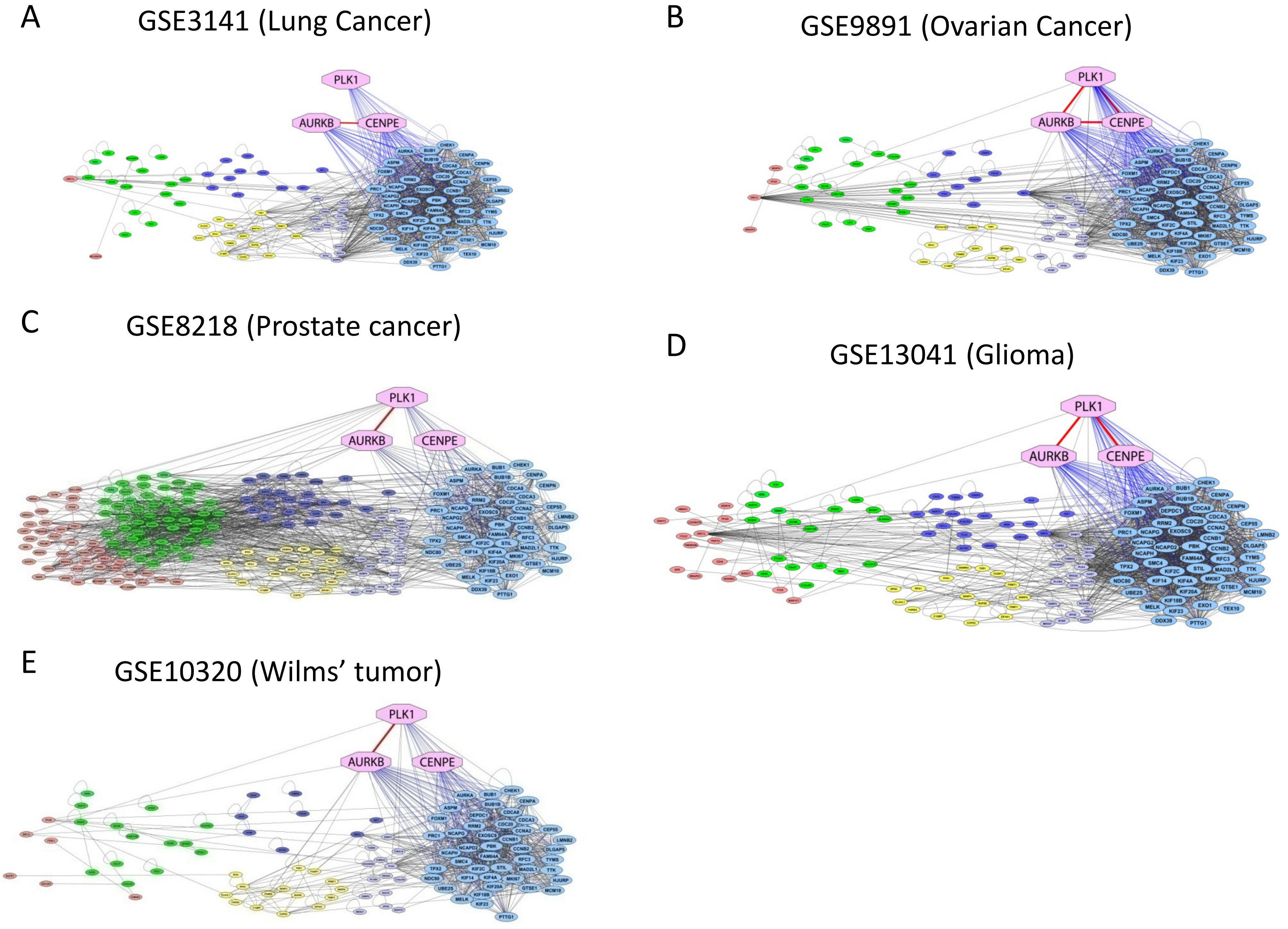

Supplemental Figure 1

F GSE12995 (Acute lymphoblastic leukemia)

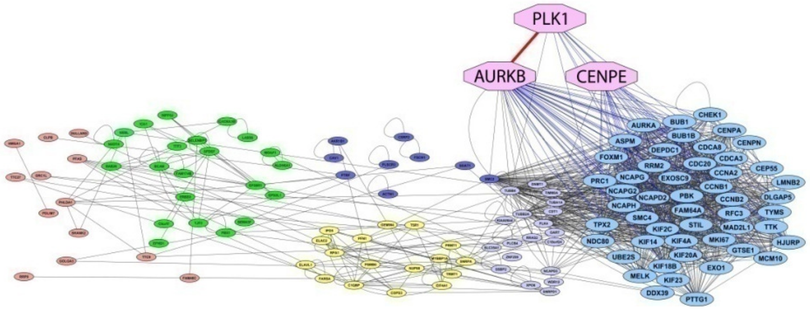

G GSE12417 (Acute Myelogenous leukemia)

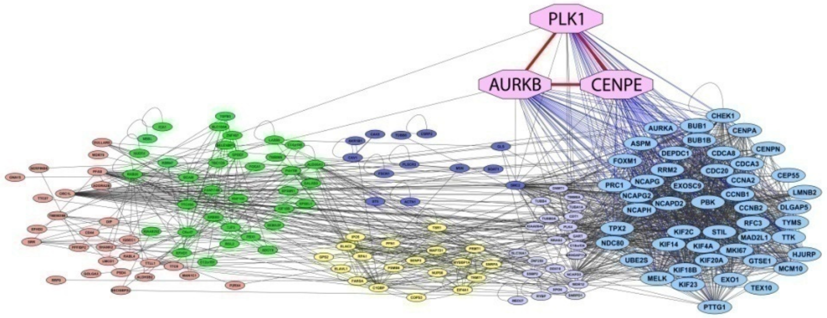

H GSE11582 (Lymphoblast Cell lines)

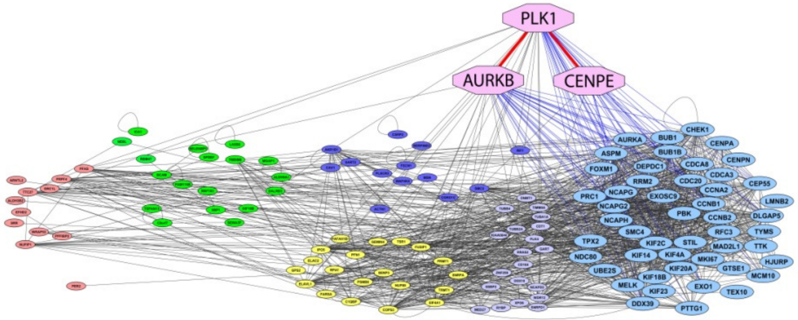

Supplemental Figure 1 (Cont.)

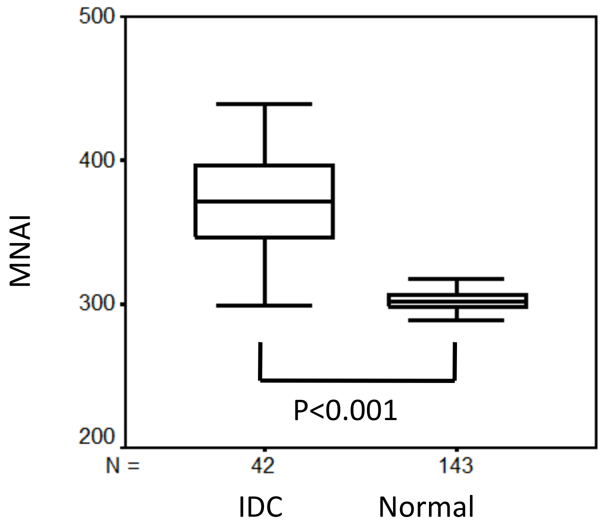

Supplemental Figure 2

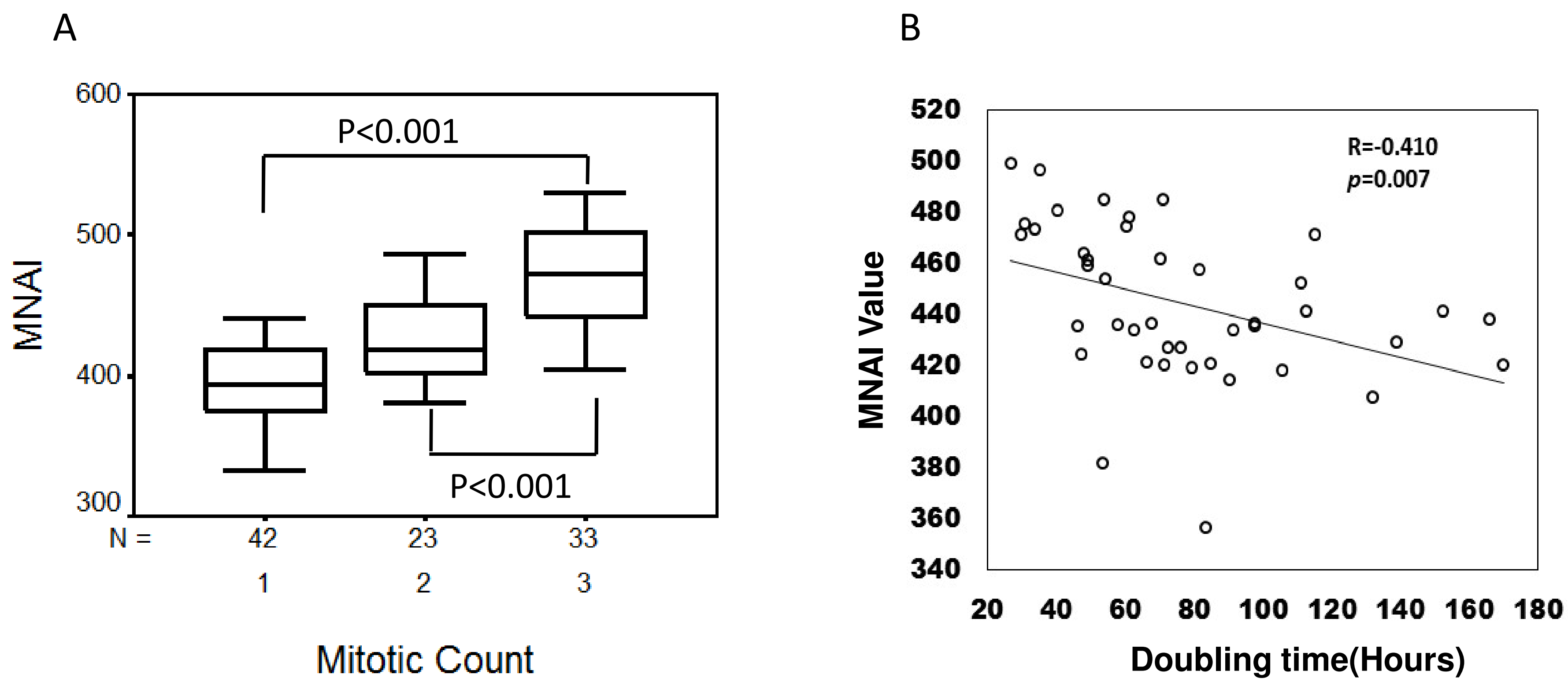

Supplemental Figure 3

C

NAME_cell line	Td(doubling time)	MNAI
MDAMB231	26.6	499.2
MCF10A	35.1	497.0
HCC1569	48.8	485.6
HCC1937	115.0	485.5
MCF12A	40.3	481.0
HS578T	60.9	478.4
SUM185PE	83.4	476.7
BT549	30.8	475.4
SUM159PT	29.8	471.4
MDAMB468	47.8	464.3
ZR75.B	70.3	462.1
MDAMB435	49.1	461.4
MDAMB436	67.5	458.7
MDAMB157	81.6	457.9
HCC1954	54.2	456.9
HCC70	60.2	454.7
HCC3153	111.0	452.2
HCC2185	165.8	450.0
HCC1428	91.3	448.1
MDAMB134	112.4	441.3
SUM52PE	53.8	441.3
MDAMB415	97.4	436.6
HCC38	57.7	436.1
MCF7	46.0	435.9
T47D	97.7	435.4
AU565	62.5	434.3
ZR75.1	138.7	429.4
SKBR3	72.2	427.1
BT474	75.9	427.0
LY2	47.1	424.6
600MPE	66.0	421.2
CAMA1	84.9	420.9
ZR75.30	169.9	420.4
HCC1500	53.4	420.0
MDAMB361	79.2	419.5
UACC812	105.4	418.3
MDAMB175	90.3	414.6
BT483	131.9	407.8
HCC1143	71.2	395.5
HCC1187	70.7	385.2
SUM149PT	33.6	383.4
SUM1315	152.3	381.6

Supplemental Figure **3**(Cont.)

A

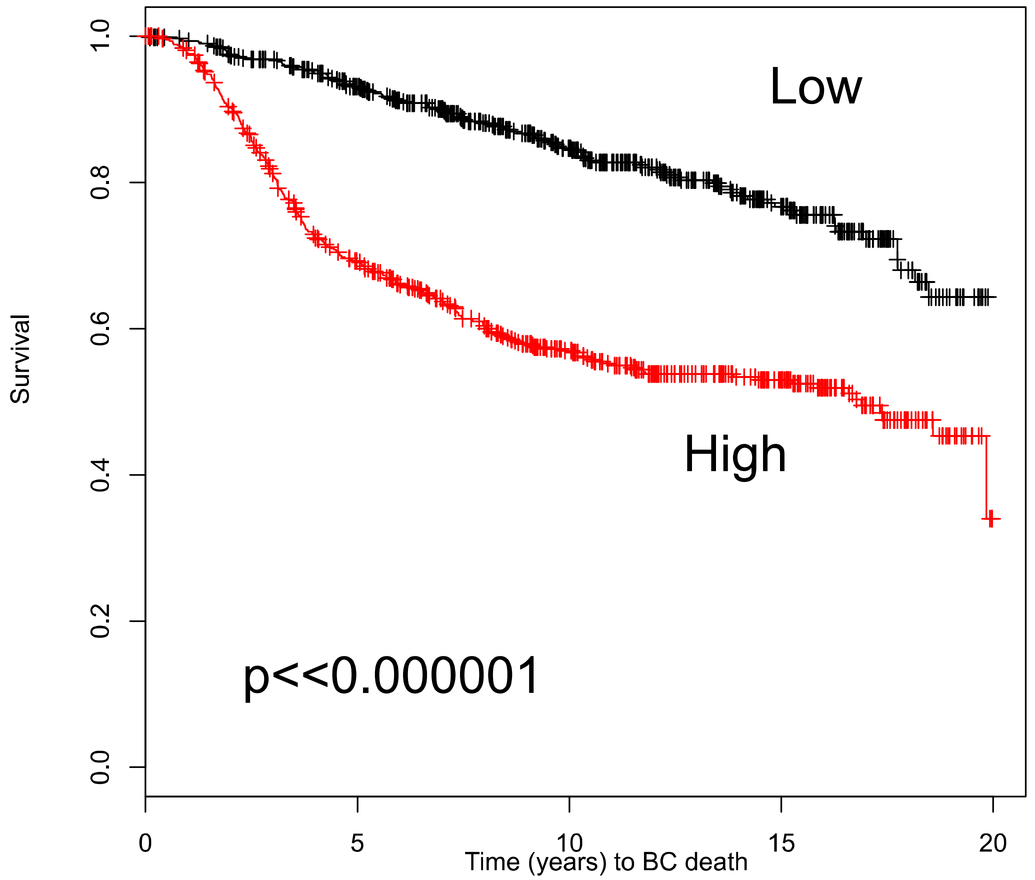

B

	X^2	DFR	P-VALUE
**MNAI**	5.64	1	0.0176
**AGE**	12.23	2	0.0022
**LYMPHNODE POSITIVE**	102.21	2	<2.2e-16
**SIZE**	15.11	2	0.0005
**GRADE**	3.12	3	0.3733
**STAGE**	16.37	5	0.0059
**PAM50**	27.782	5	3.9e-5

Supplemental Figure 4

A

B

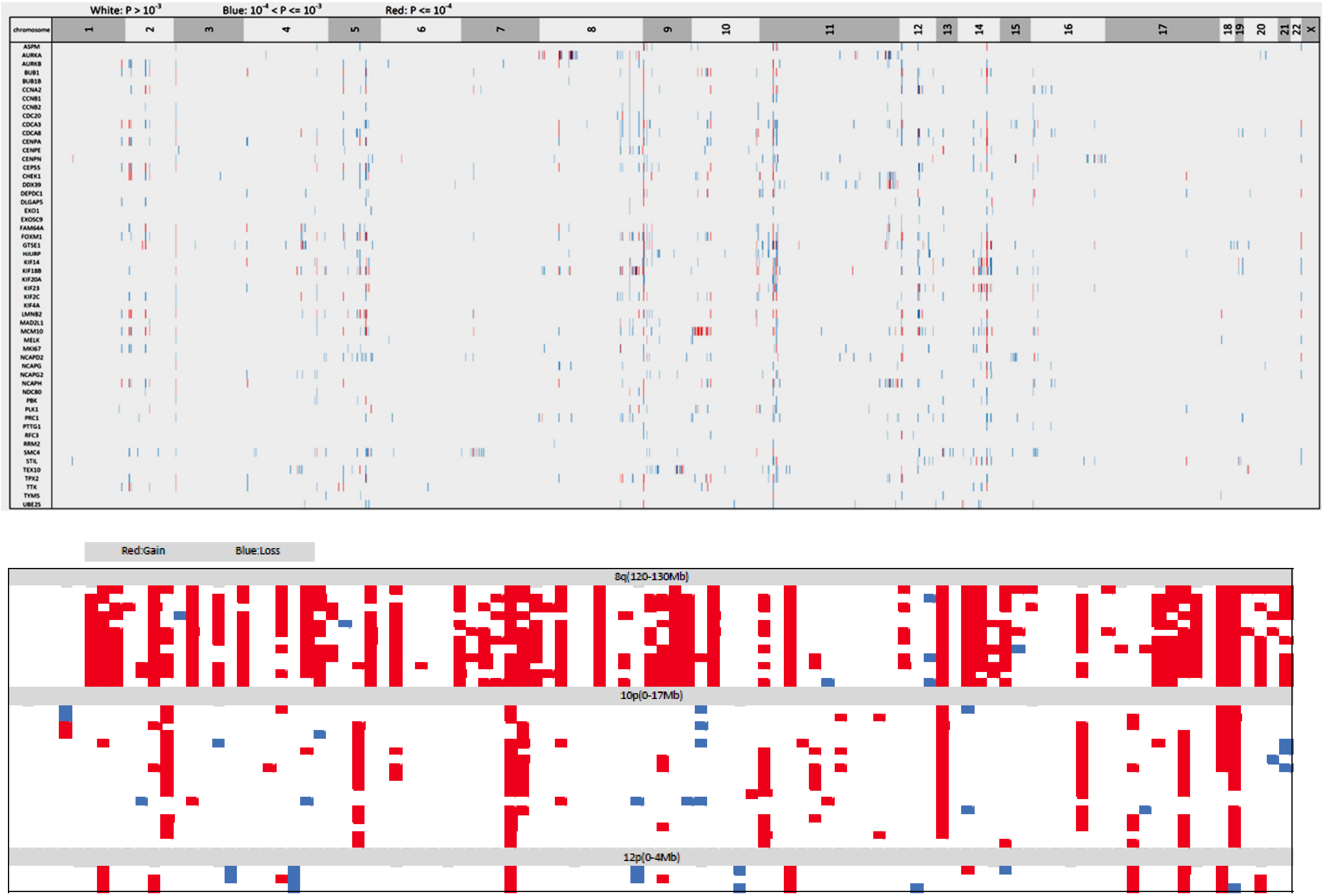

Supplemental Figure 5

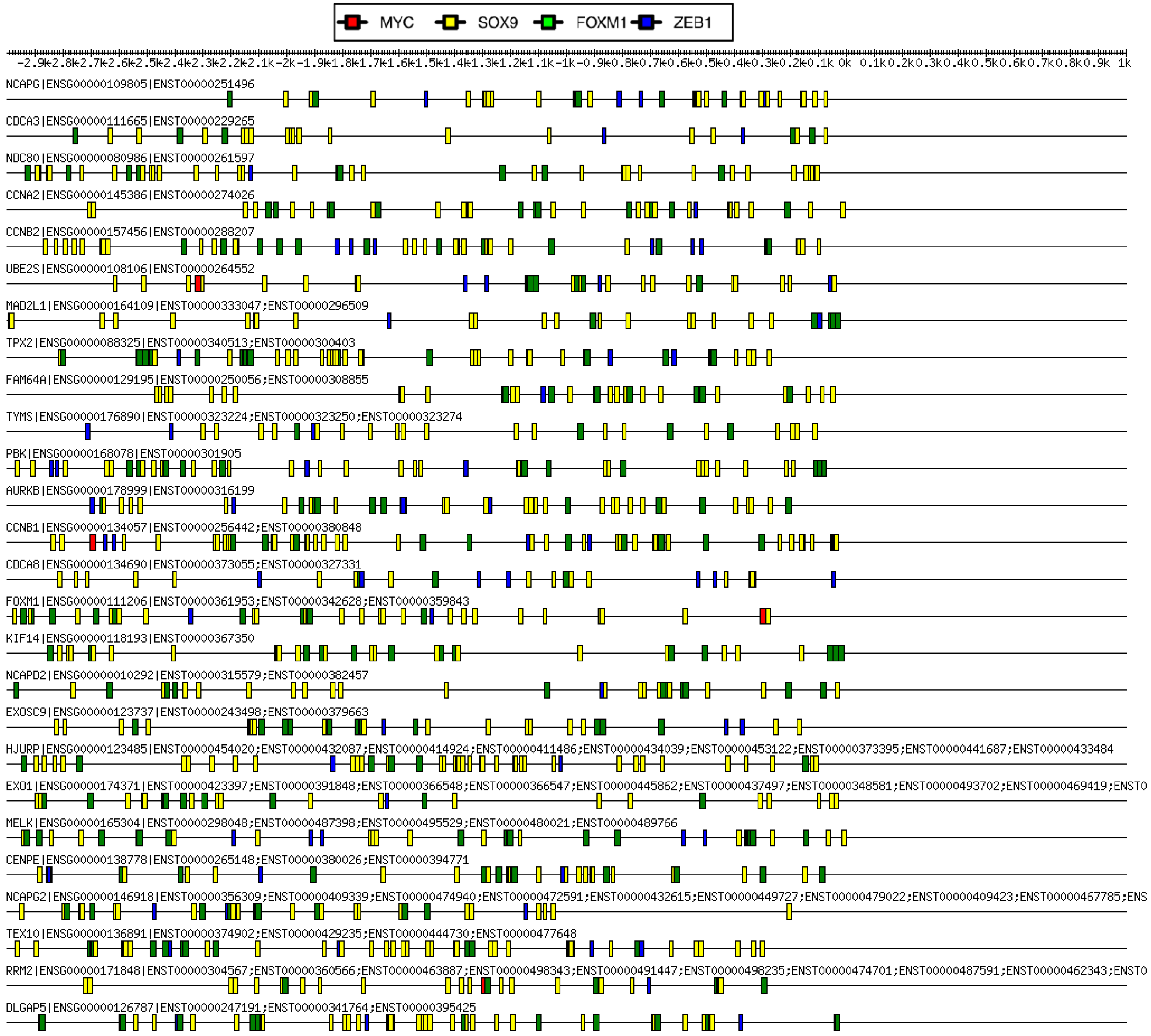

Supplemental Figure 7

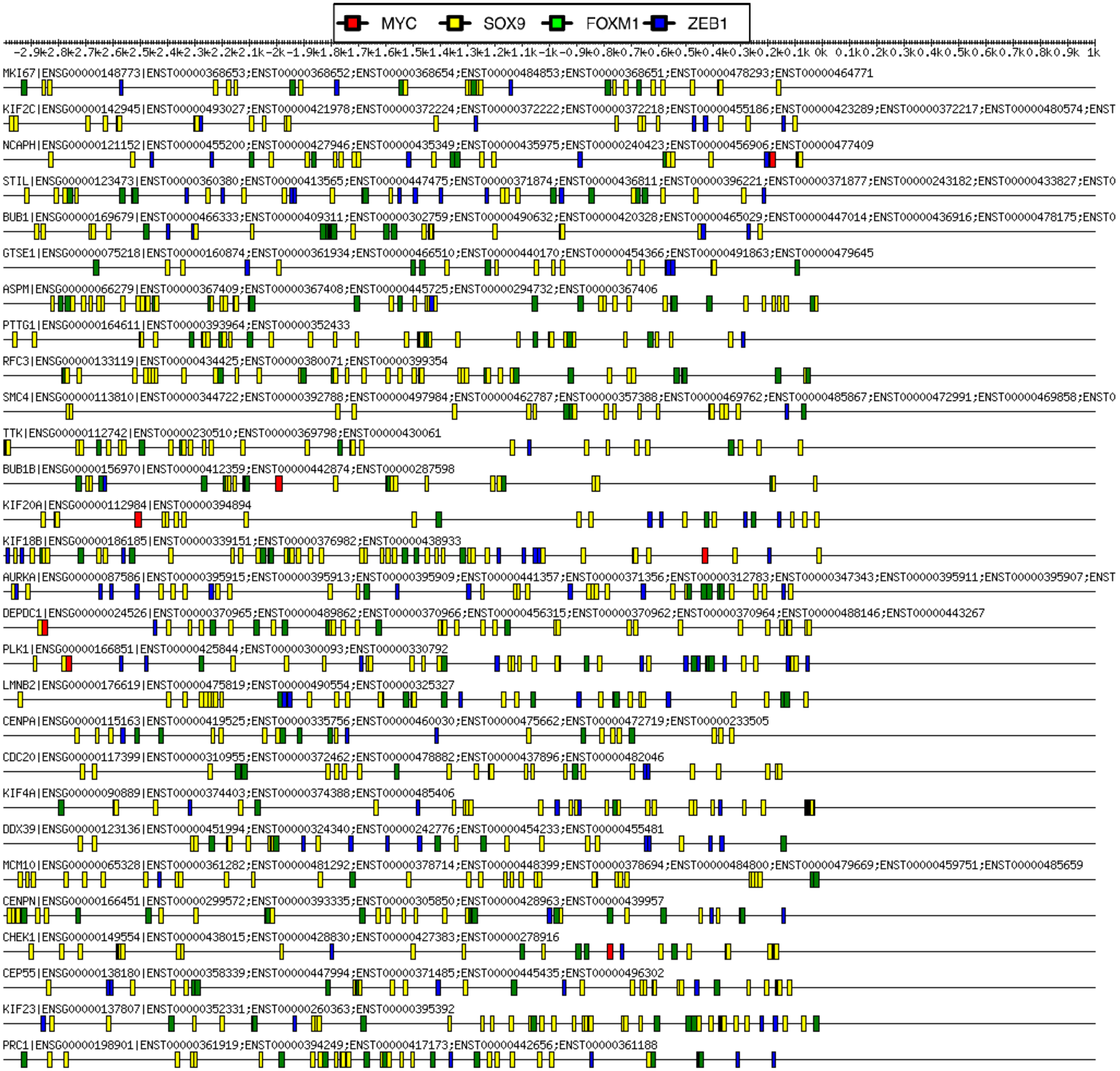

Supplemental Figure 7 (Cont.)

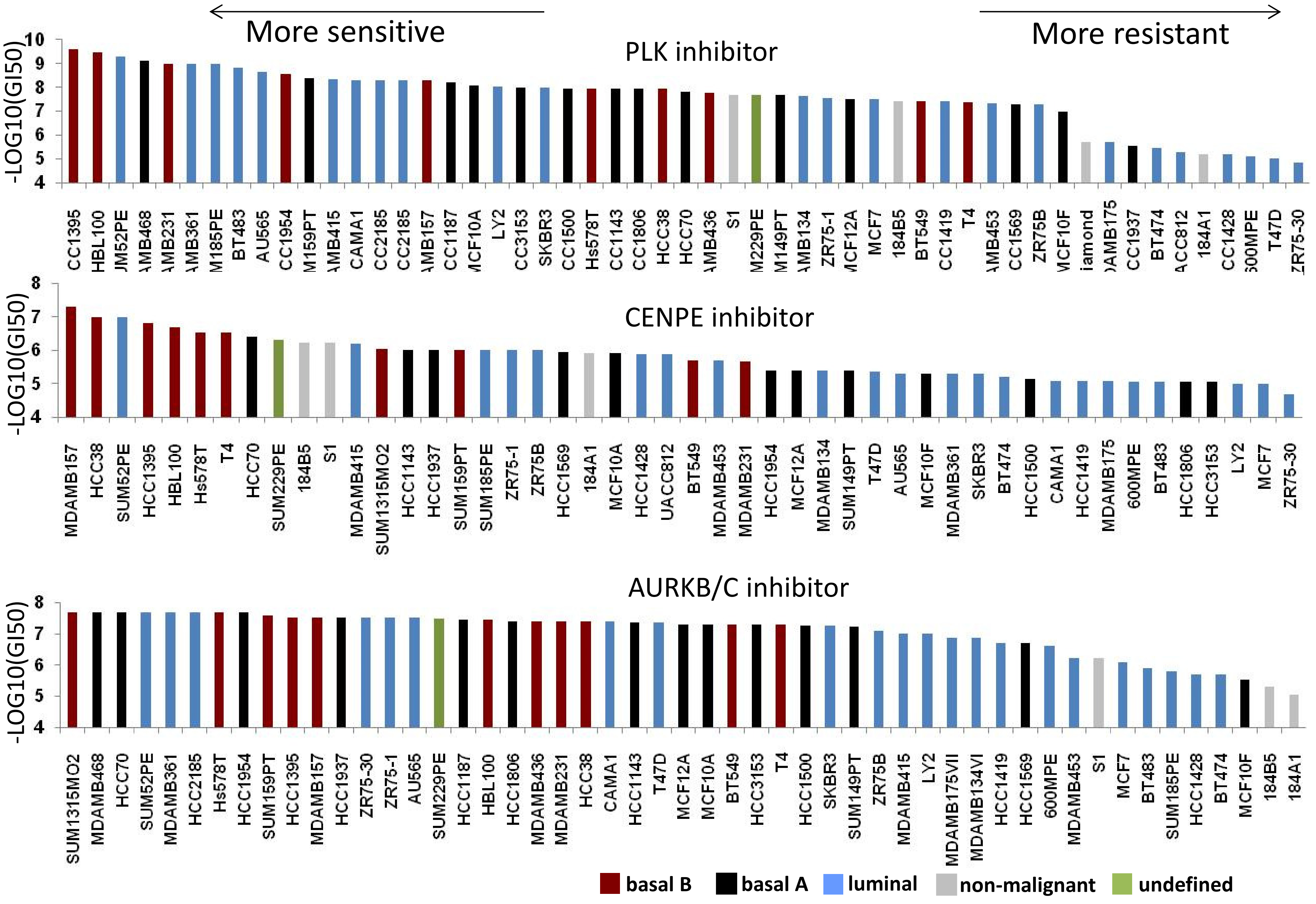

Supplemental Figure 8

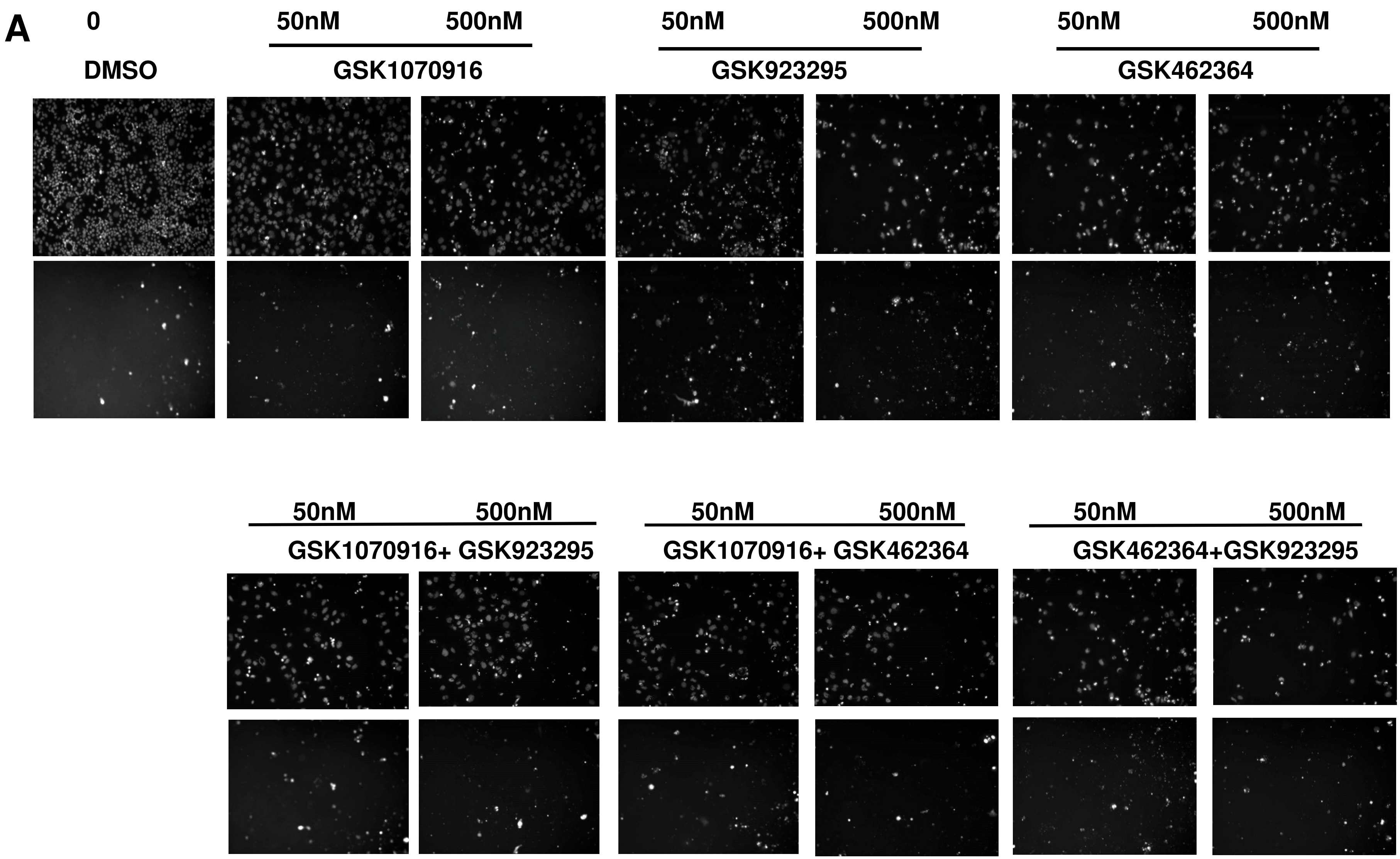

Supplemental Figure 9

B

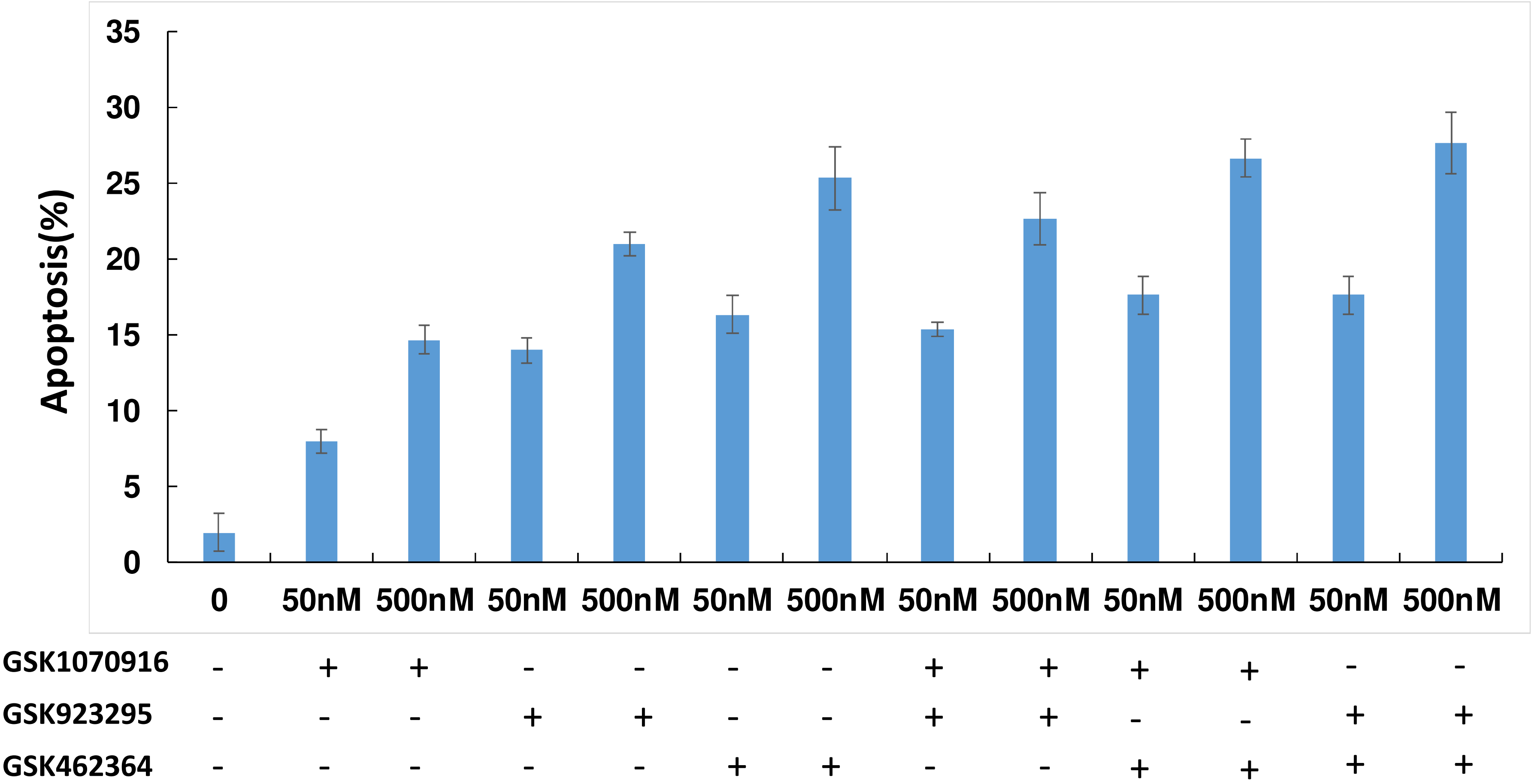

Supplemental Figure 9 (Cont.)

Reference

Bild, A.H., Yao, G., Chang, J.T., Wang Q., Potti, A., Chasse, D., Joshi, M.B., Harpole, D., Lancaster, J.M., Berchuck, A., et al. (2006). Oncogenic pathway signatures in human cancers as a guide to targeted therapies. Nature 439, 353-357.

Huang, C.C., Gadd, S., Breslow, N., Cutcliffe, C., Sredni, S.T., Helenowski, I.B., Dome, J.S., Grundy, P.E., Green, D.M., Fritsch, M.K., et al. (2009). Predicting relapse in favorable histology Wilms' tumor using gene expression analysis: a report from the Renal Tumor Committee of the Children's Oncology Group. Clin. Cancer Res. 15,1770-8.

Lee, Y., Scheck, A.C., Cloughesy, T.F., Lai, A., Dong, J., Farooqi, H.K., Liau, L.M., Horvath, S., Mischel, P.S., Nelson, S.F. et al. Gene expression analysis of glioblastomas identifies the major molecular basis for the prognostic benefit of younger age. BMC. Med. Genomics 1, 52 (2008).

Metzeler, K.H., Hummel, M., Bloomfield, C.D., Spiekermann, K., Braess ,J., Sauerland, M.C., Heinecke, A., Radmacher, M., Marcucci, G., Whitman, S.P., et al. (2008). An 86-probe-set gene-expression signature predicts survival in cytogenetically normal acute myeloid leukemia. Blood 112, 4193-201.

Choy, E., Yelensky, R., Bonakdar, S., Plenge, R.M., Saxena, R., De Jager, P.L., Shaw, S.Y., Wolfish, C.S., Slavik, J.M., Cotsapas, C., et al. (2008). Genetic analysis of human traits in vitro: drug response and gene expression in lymphoblastoid cell lines. PLoS Genet. 4, e1000287.
