# Supplementary material for: Genome co-amplification upregulates a mitotic gene network activity that predicts outcome and response to mitotic protein inhibitors in breast cancer
Source: Breast Cancer Res. 2016 Jul 1;18:70. doi: 10.1186/s13058-016-0728-y (PMC4930593; doi:10.1186/s13058-016-0728-y)
Supplement: Additional file 4: Figure S1. — Manhattan plots for each mitotic network gene illustrating the strength of association (-log10 p value) between the expression of the gene and genome-wide somatic copy number alterations (see Fig S6). (PDF 8516 kb) [file 13058_2016_728_MOESM6_ESM.pdf]

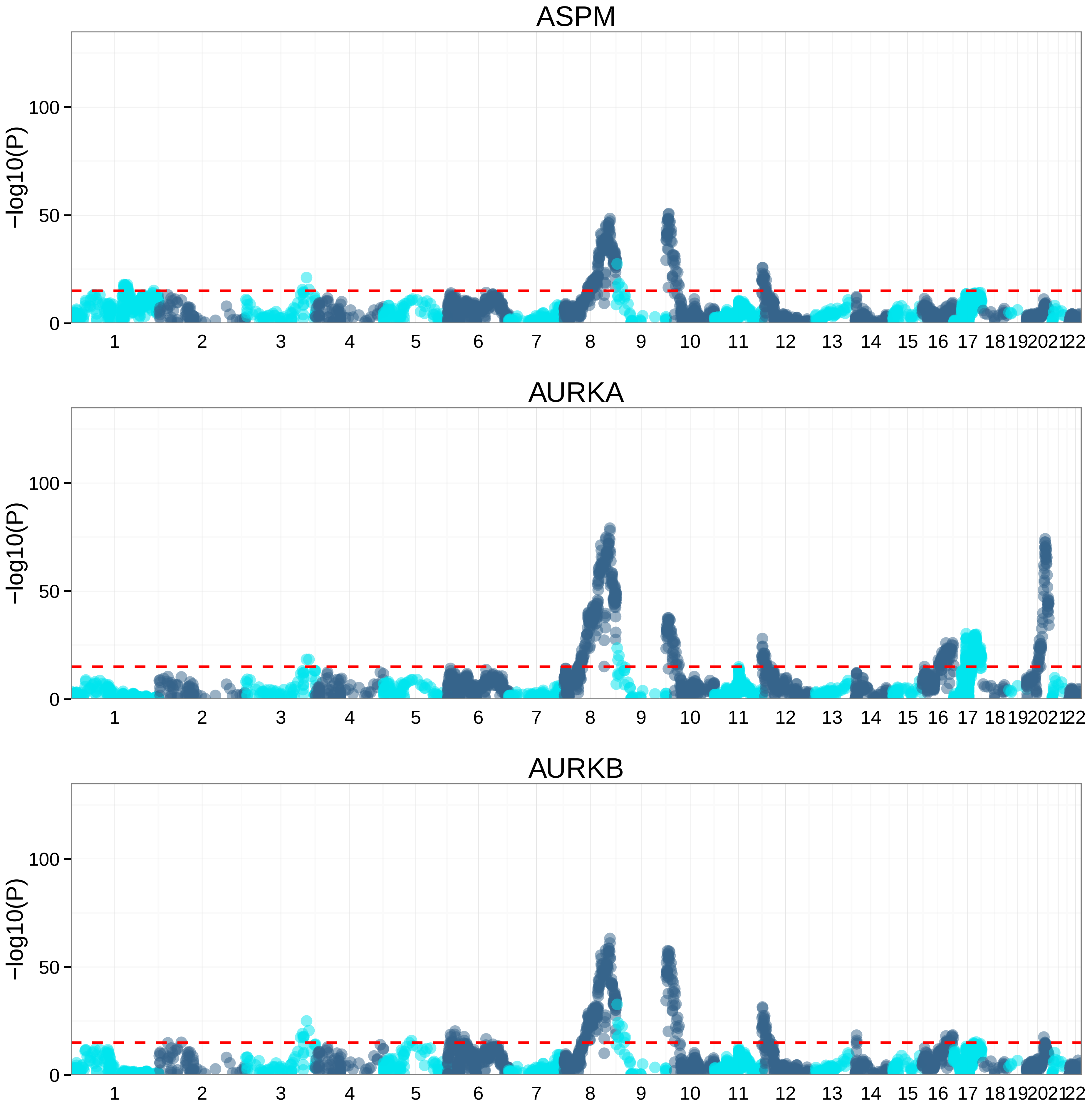
ASPM
AURKA
AURKB
−log10(P)
0
50
100
1 2 3 4 5 6 7 8 9 10 11 12 13 14 15 16 17 18 19 20 21 22

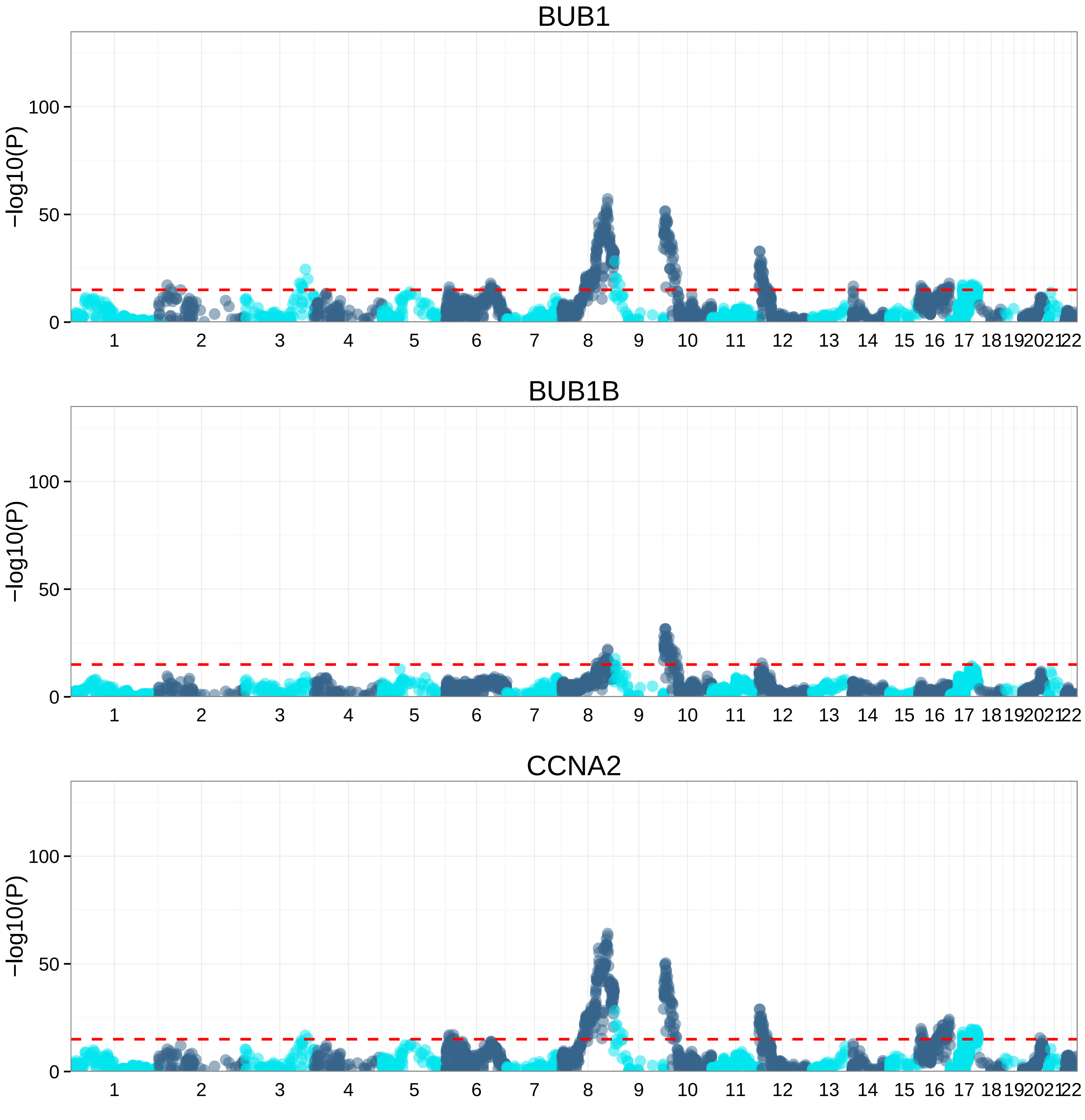
BUB1
BUB1B
CCNA2
−log10(P)
100
50
0
1
2
3
4
5
6
7
8
9
10
11
12
13
14
15
16
17
18
19
20
21
22

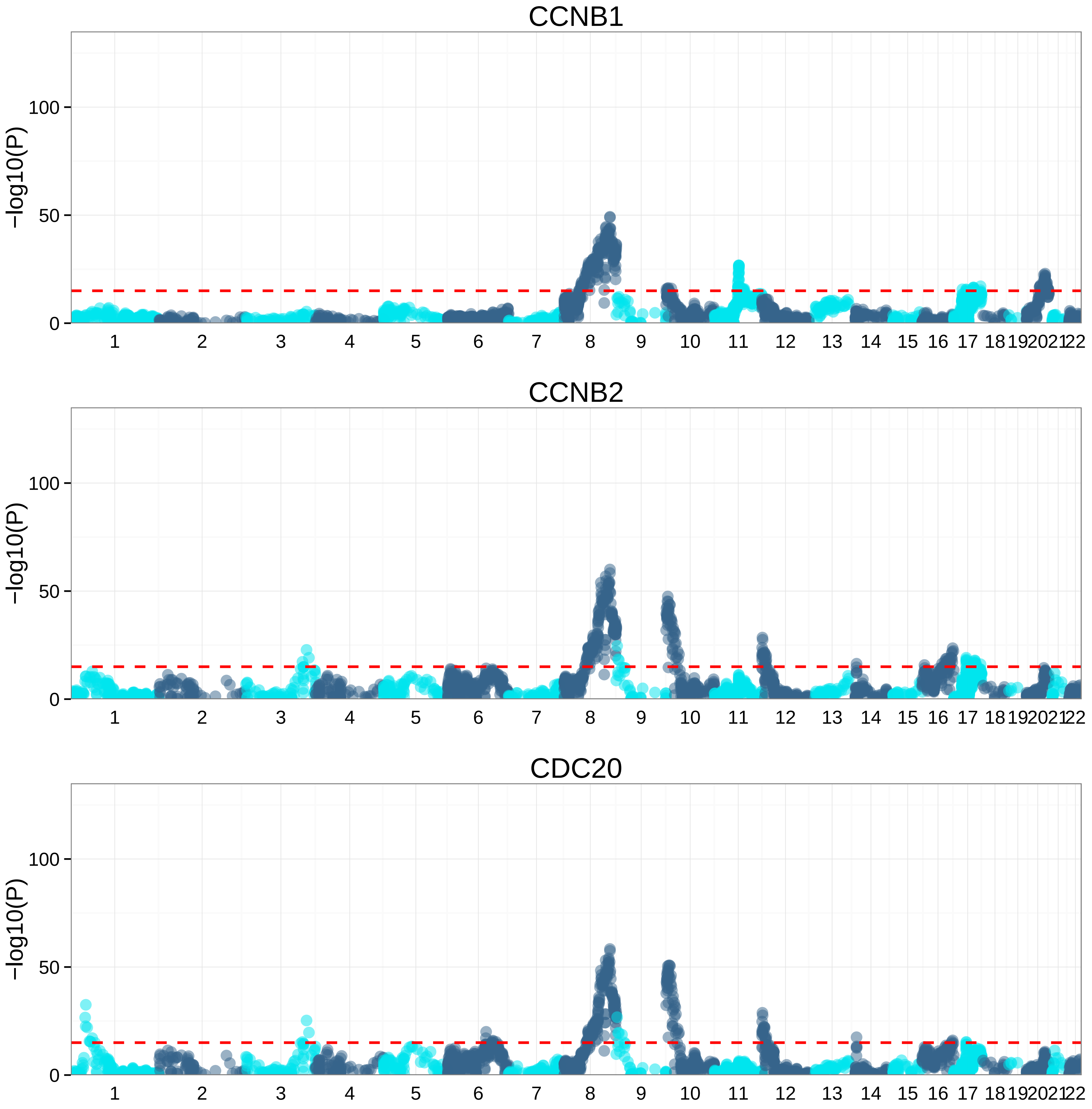

CCNB1
−log10(P)
0
50
100
1
2
3
4
5
6
7
8
9
10
11
12
13
14
15
16
17
18
19
20
21
22
CCNB2
−log10(P)
0
50
100
1
2
3
4
5
6
7
8
9
10
11
12
13
14
15
16
17
18
19
20
21
22
CDC20
−log10(P)
0
50
100
1
2
3
4
5
6
7
8
9
10
11
12
13
14
15
16
17
18
19
20
21
22

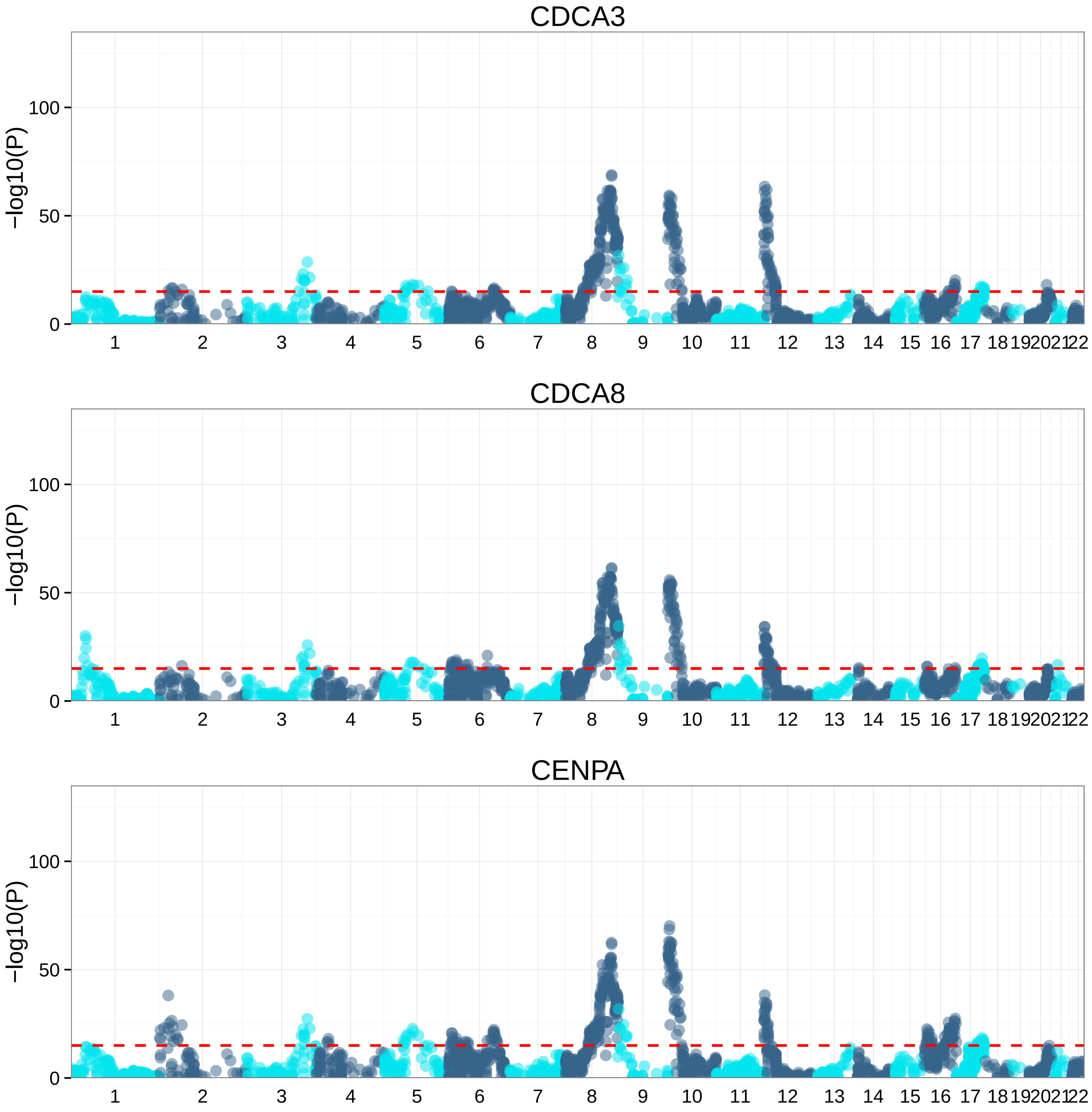
CDCA3
−log10(P)
100
50
0
1 2 3 4 5 6 7 8 9 10 11 12 13 14 15 16 17 18 19 20 21 22
CDCA8
−log10(P)
100
50
0
1 2 3 4 5 6 7 8 9 10 11 12 13 14 15 16 17 18 19 20 21 22
CENPA
−log10(P)
100
50
0
1 2 3 4 5 6 7 8 9 10 11 12 13 14 15 16 17 18 19 20 21 22

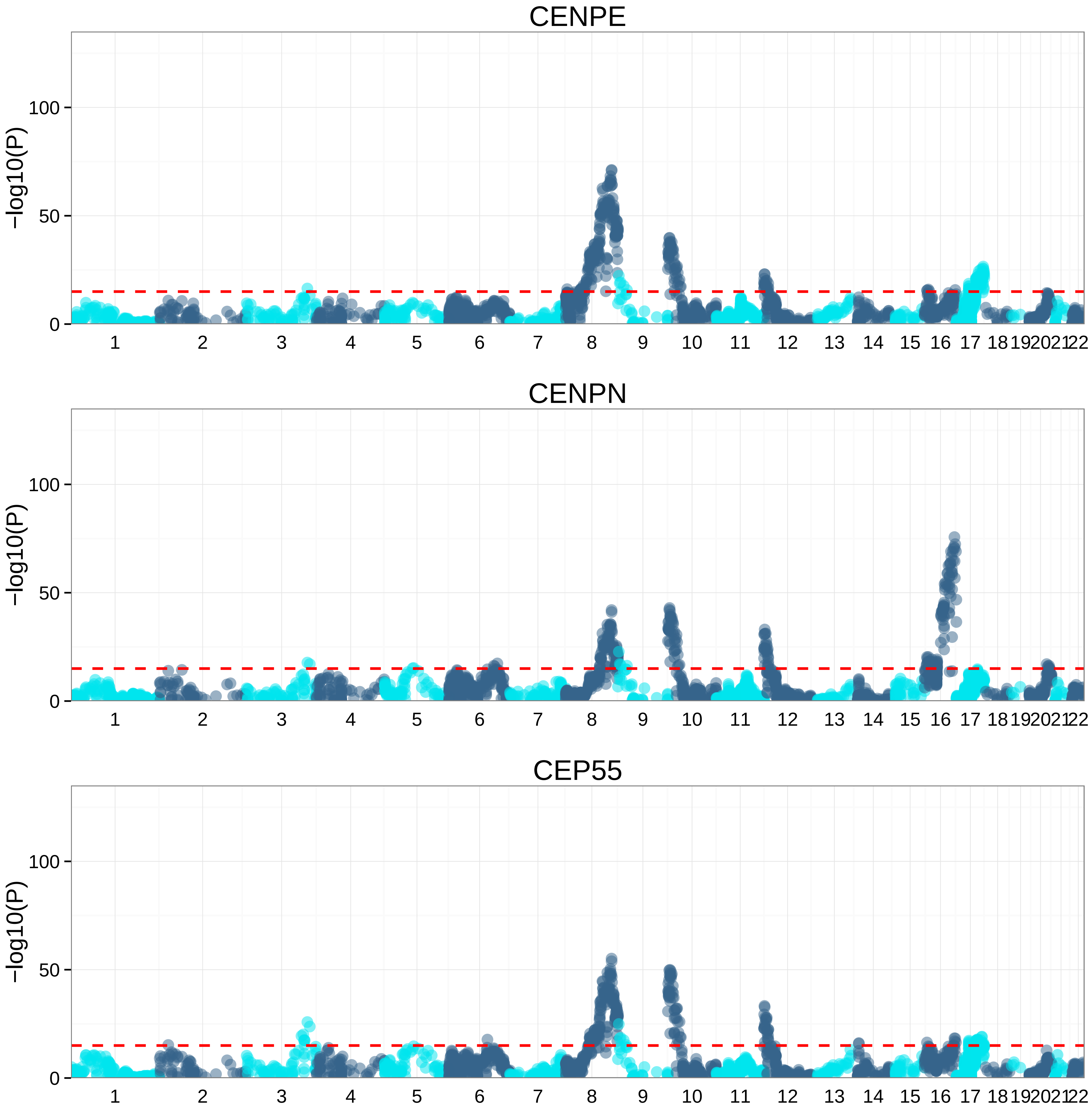
CENPE
−log10(P)
100
50
0
1
2
3
4
5
6
7
8
9
10
11
12
13
14
15
16
17
18
19
20
21
22
CENPN
−log10(P)
100
50
0
1
2
3
4
5
6
7
8
9
10
11
12
13
14
15
16
17
18
19
20
21
22
CEP55
−log10(P)
100
50
0
1
2
3
4
5
6
7
8
9
10
11
12
13
14
15
16
17
18
19
20
21
22

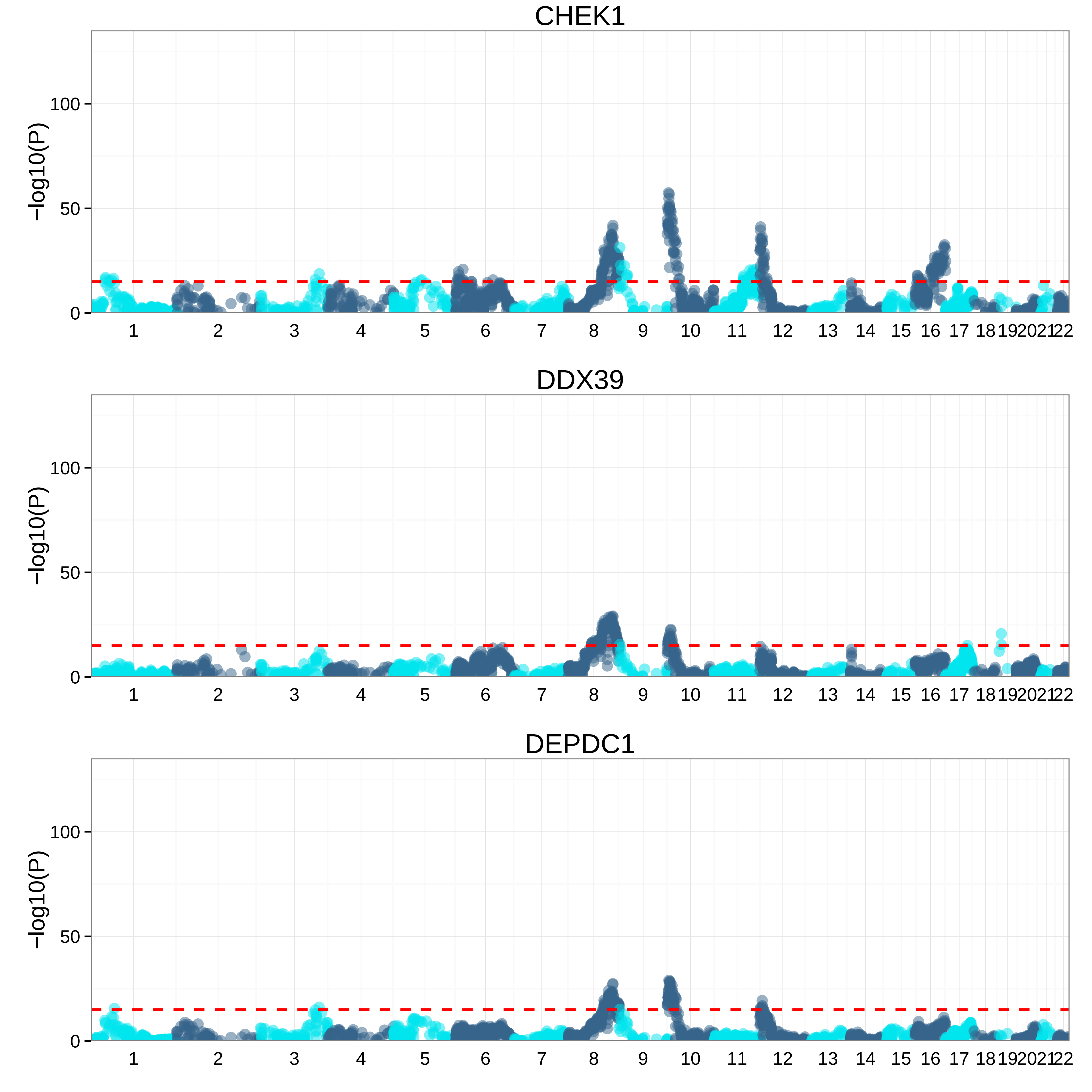
CHEK1
−log10(P)
0
50
100
1 2 3 4 5 6 7 8 9 10 11 12 13 14 15 16 17 18 19 20 21 22
DDX39
−log10(P)
0
50
100
1 2 3 4 5 6 7 8 9 10 11 12 13 14 15 16 17 18 19 20 21 22
DEPDC1
−log10(P)
0
50
100
1 2 3 4 5 6 7 8 9 10 11 12 13 14 15 16 17 18 19 20 21 22

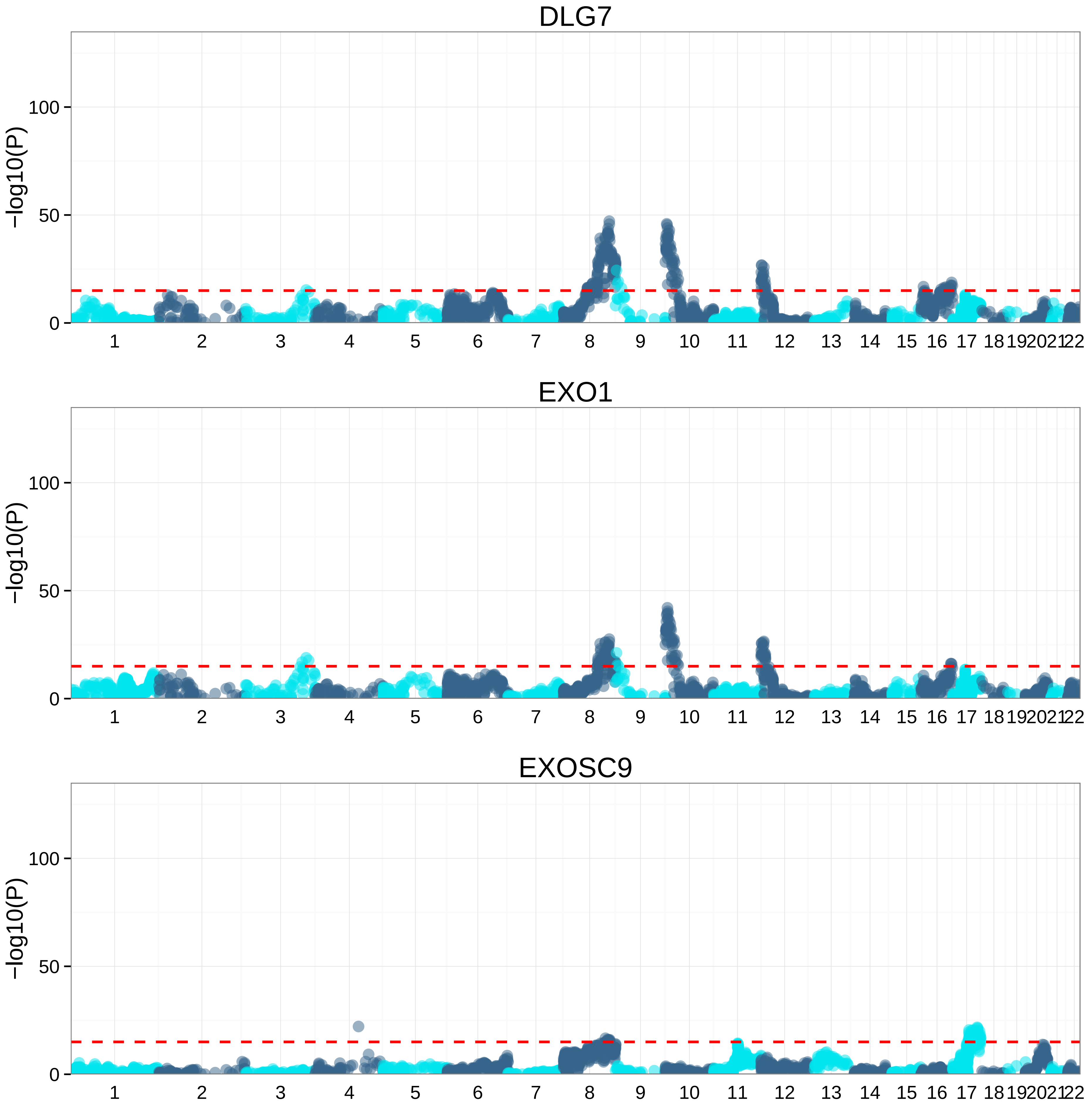
DLG7
EXO1
EXOSC9
−log10(P)
0
50
100
1 2 3 4 5 6 7 8 9 10 11 12 13 14 15 16 17 18 19 20 21 22

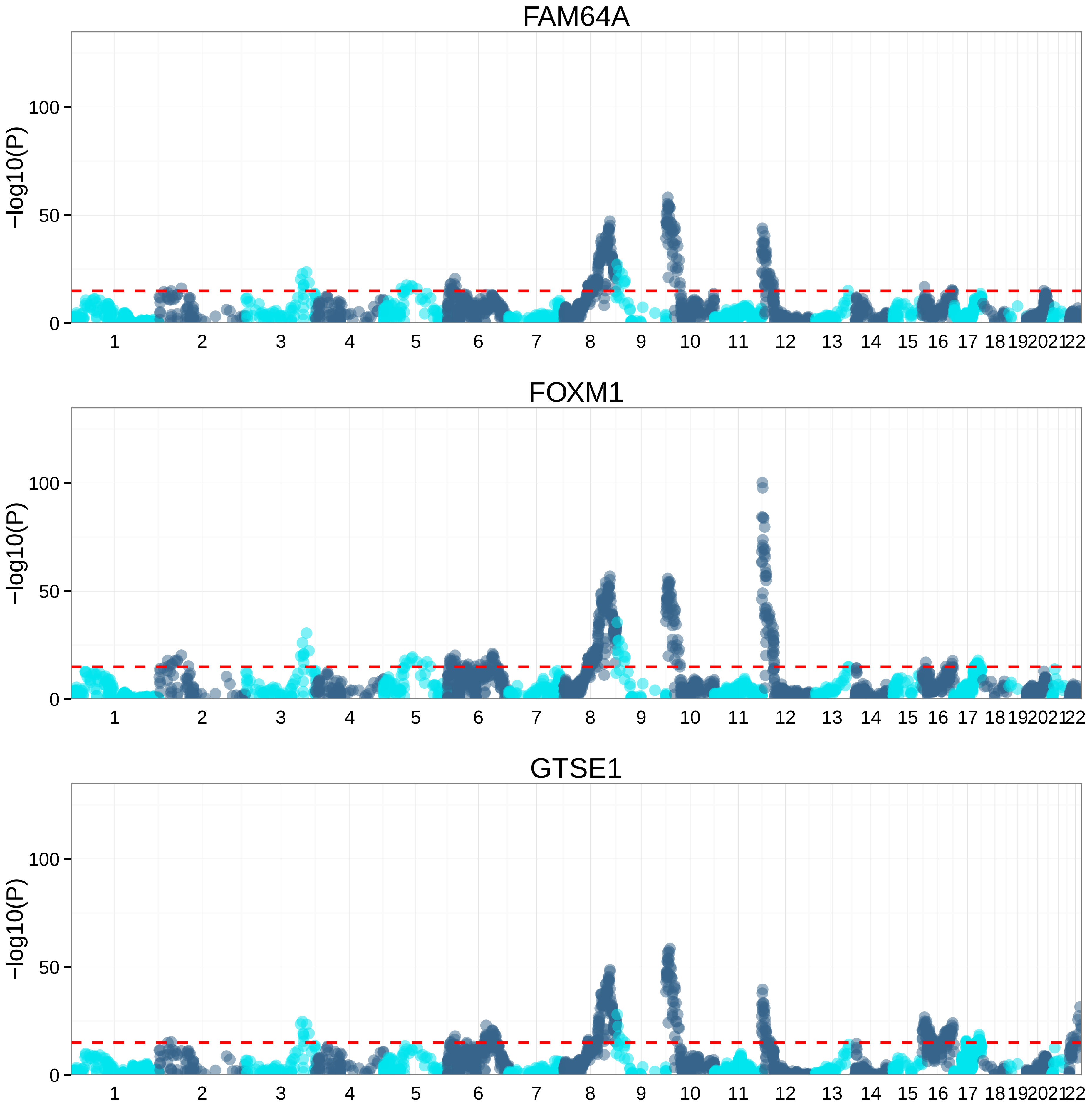
FAM64A
FOXM1
GTSE1
−log10(P)
0
50
100
1
2
3
4
5
6
7
8
9
10
11
12
13
14
15
16
17
18
19
20
21
22

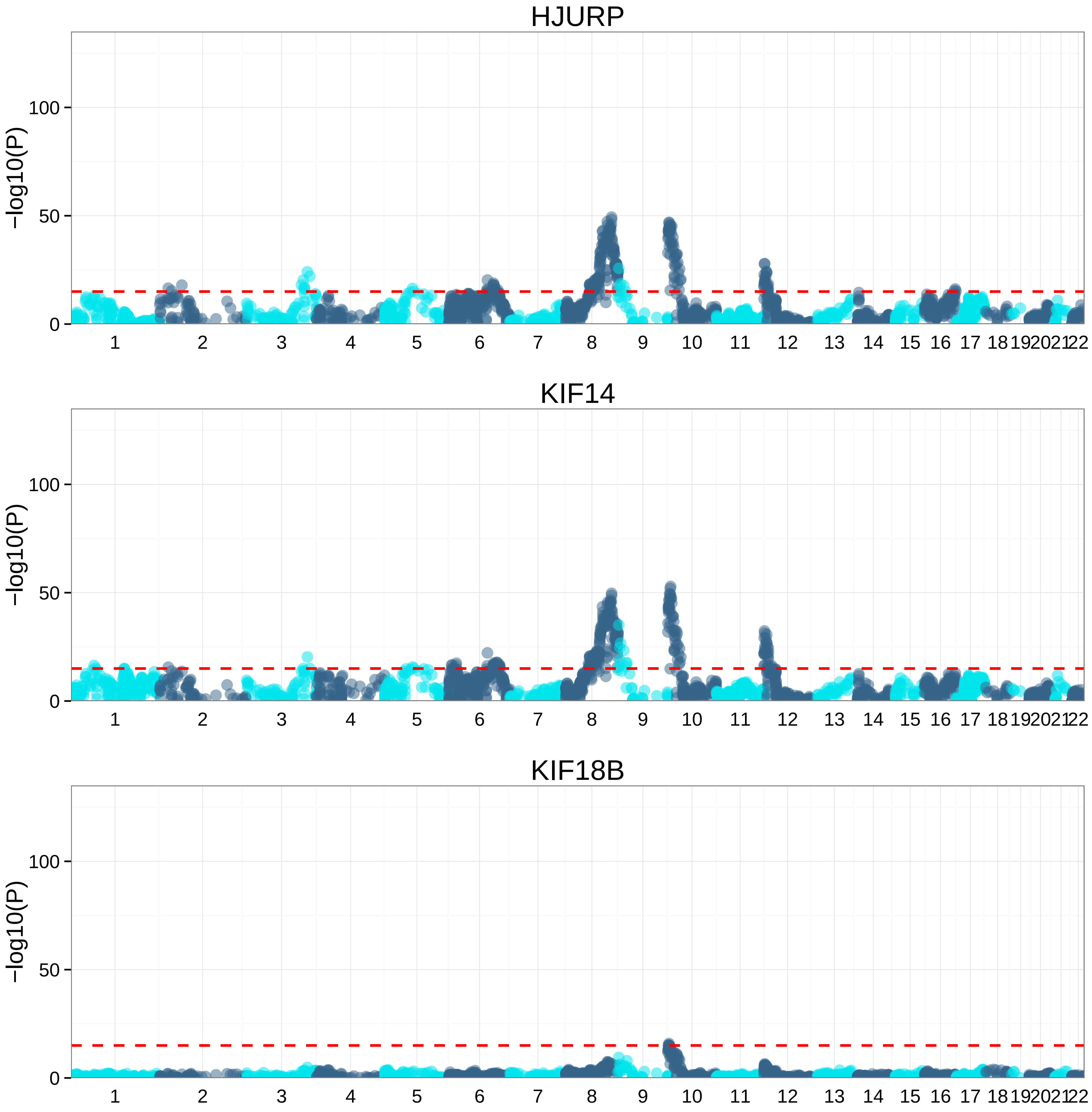
HJURP
−log10(P)
0
50
100
1 2 3 4 5 6 7 8 9 10 11 12 13 14 15 16 17 18 19 20 21 22
KIF14
−log10(P)
0
50
100
1 2 3 4 5 6 7 8 9 10 11 12 13 14 15 16 17 18 19 20 21 22
KIF18B
−log10(P)
0
50
100
1 2 3 4 5 6 7 8 9 10 11 12 13 14 15 16 17 18 19 20 21 22

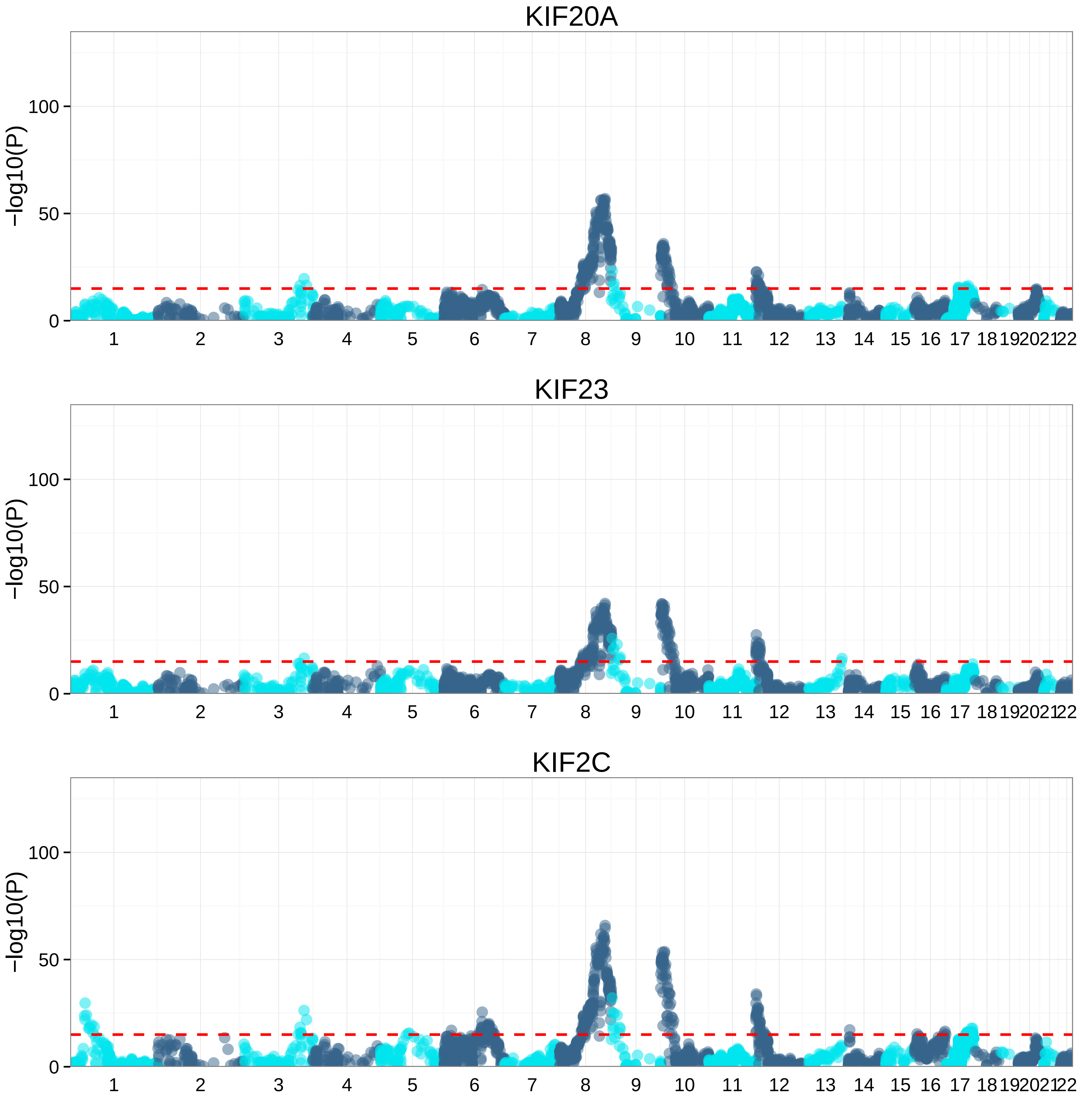
KIF20A
KIF23
KIF2C
−log10(P)
0
50
100
1
2
3
4
5
6
7
8
9
10
11
12
13
14
15
16
17
18
19
20
21
22

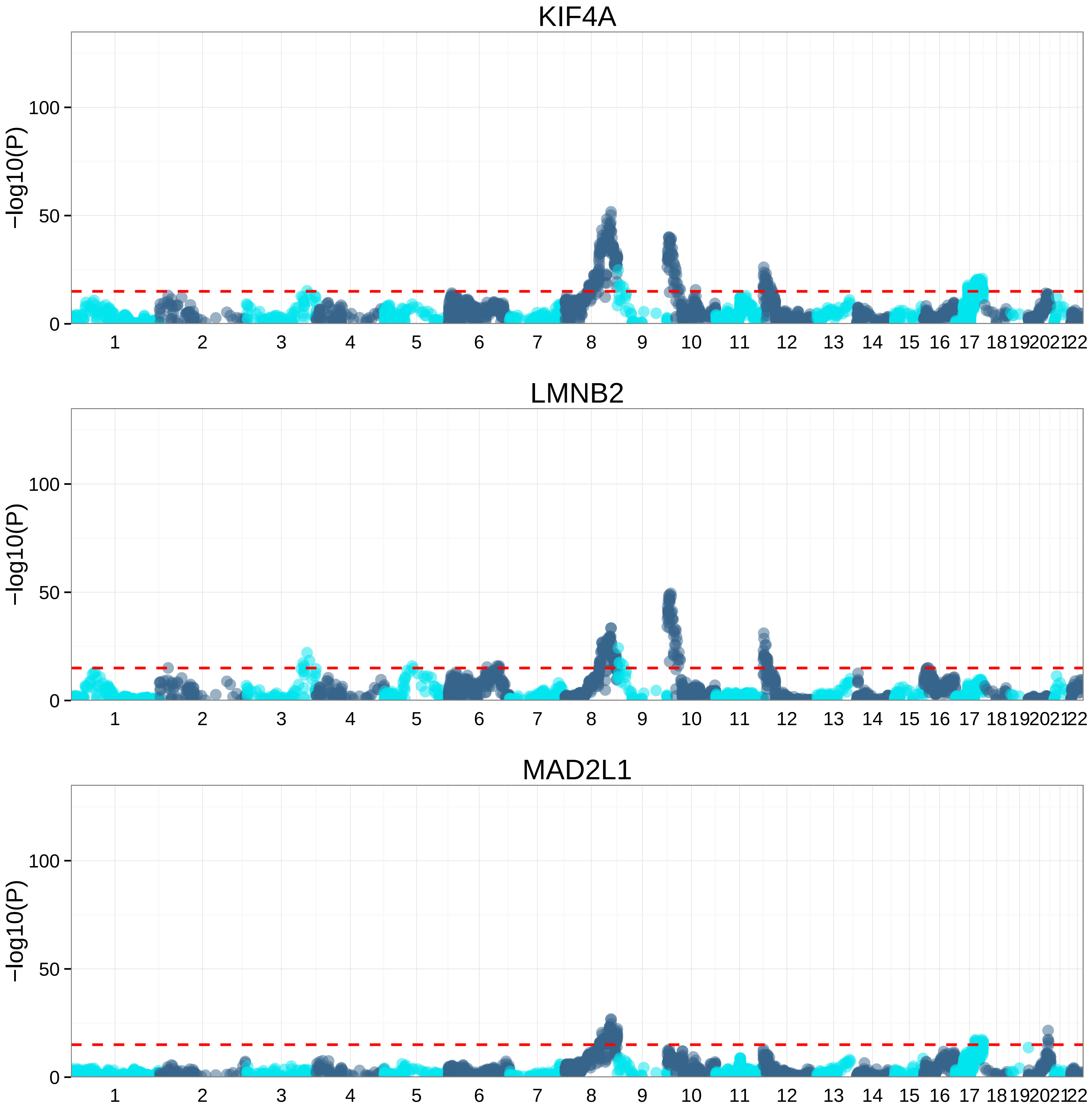
KIF4A
LMNB2
MAD2L1
−log10(P)
0
50
100
1 2 3 4 5 6 7 8 9 10 11 12 13 14 15 16 17 18 19 20 21 22

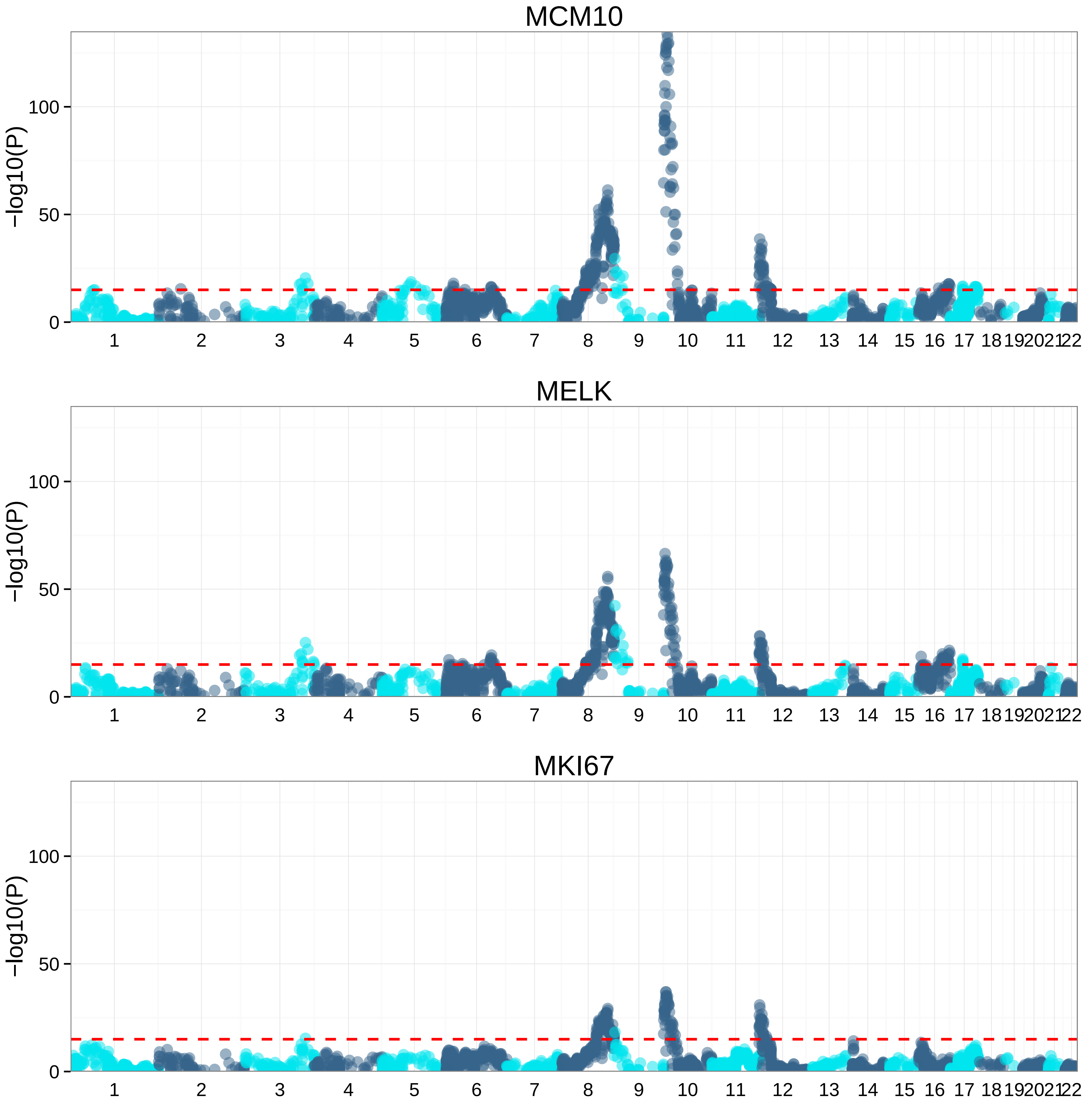
MCM10
MELK
MKI67
−log10(P)
0
50
100
1
2
3
4
5
6
7
8
9
10
11
12
13
14
15
16
17
18
19
20
21
22

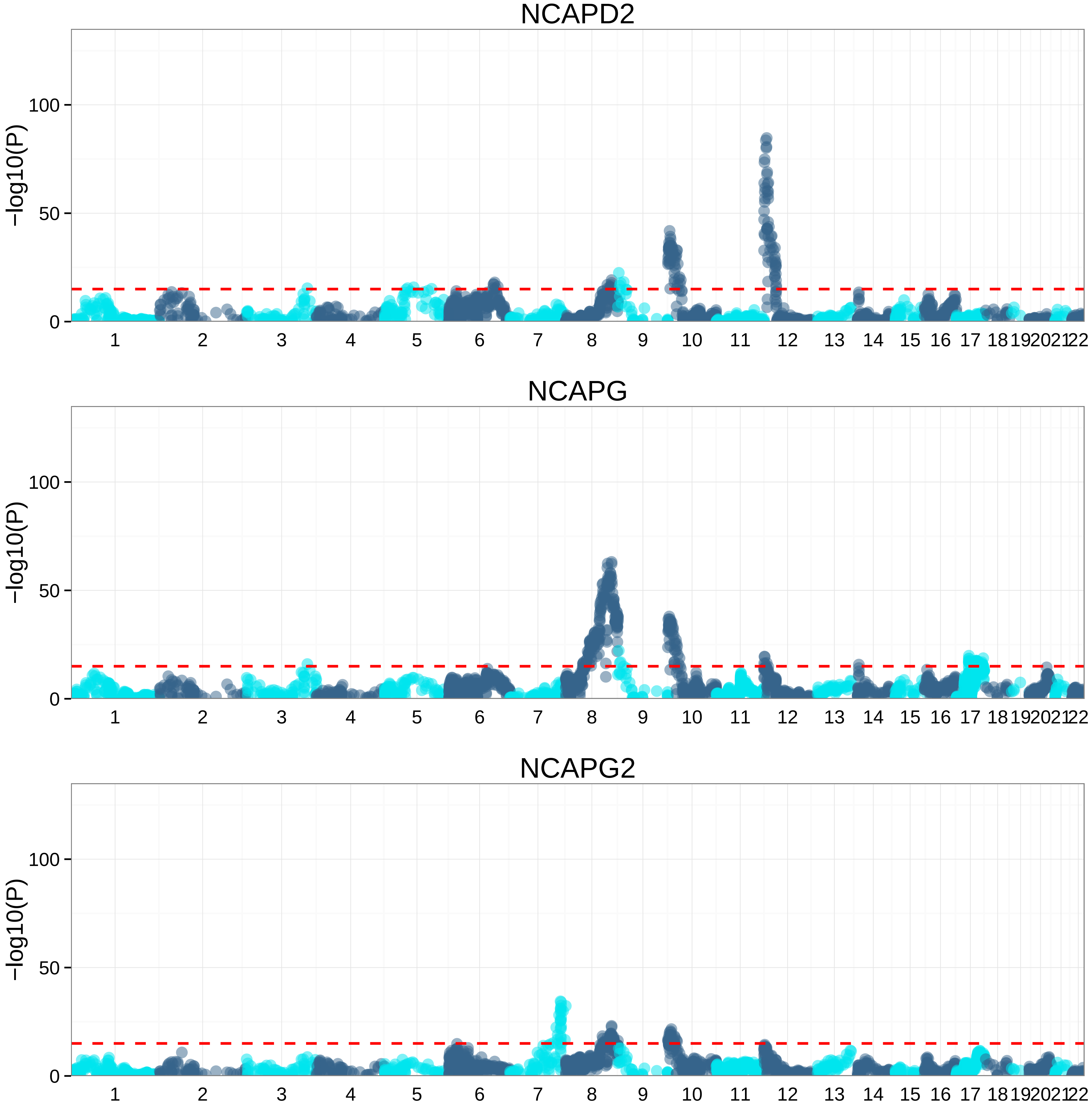
NCAPD2
NCAPG
NCAPG2
−log10(P)
0
50
100
1
2
3
4
5
6
7
8
9
10
11
12
13
14
15
16
17
18
19
20
21
22

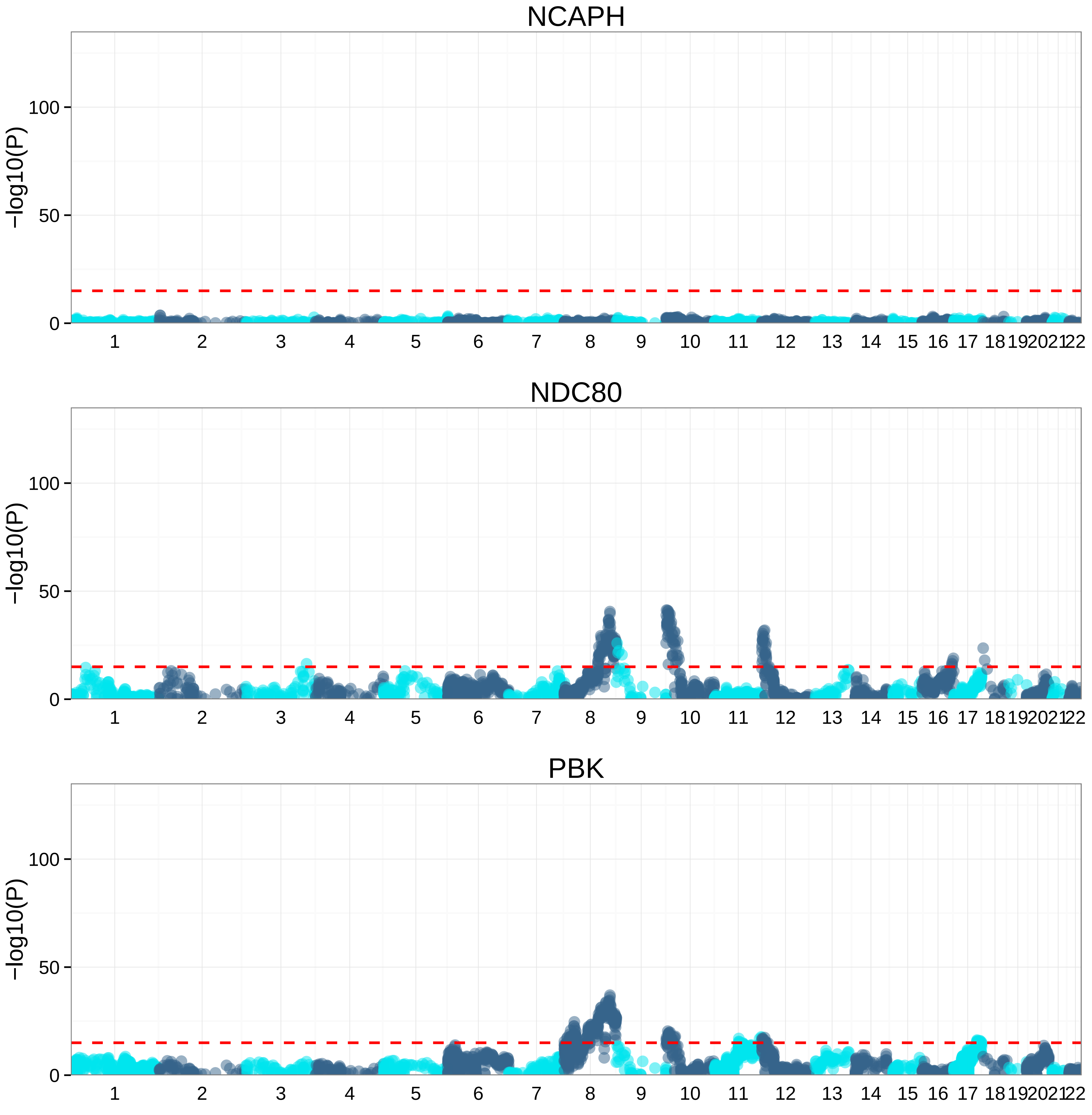

NCAPH
NDC80
PBK
−log10(P)
0
50
100
1
2
3
4
5
6
7
8
9
10
11
12
13
14
15
16
17
18
19
20
21
22

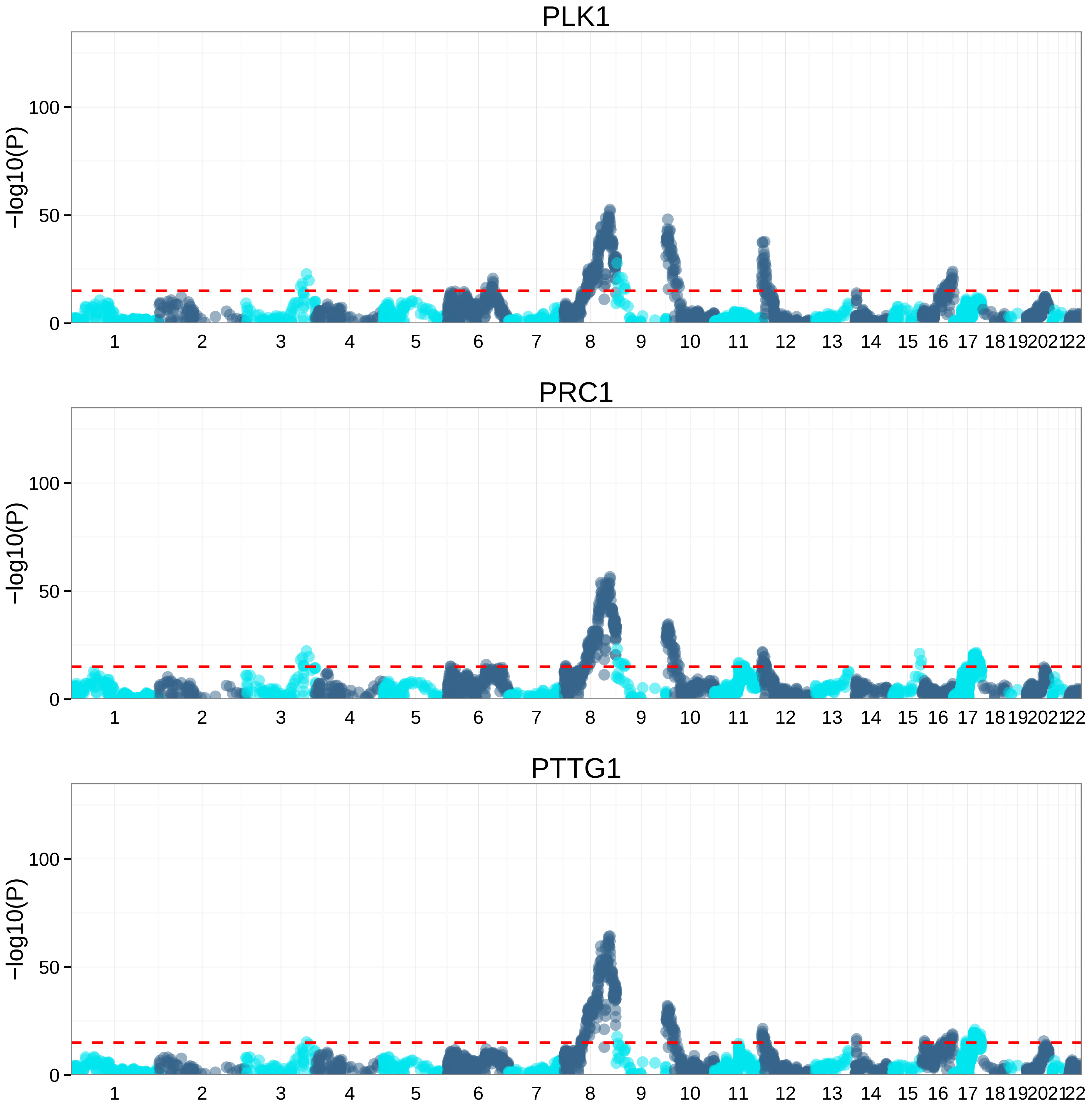
PLK1
PRC1
PTTG1
−log10(P)
0
50
100
1 2 3 4 5 6 7 8 9 10 11 12 13 14 15 16 17 18 19 20 21 22

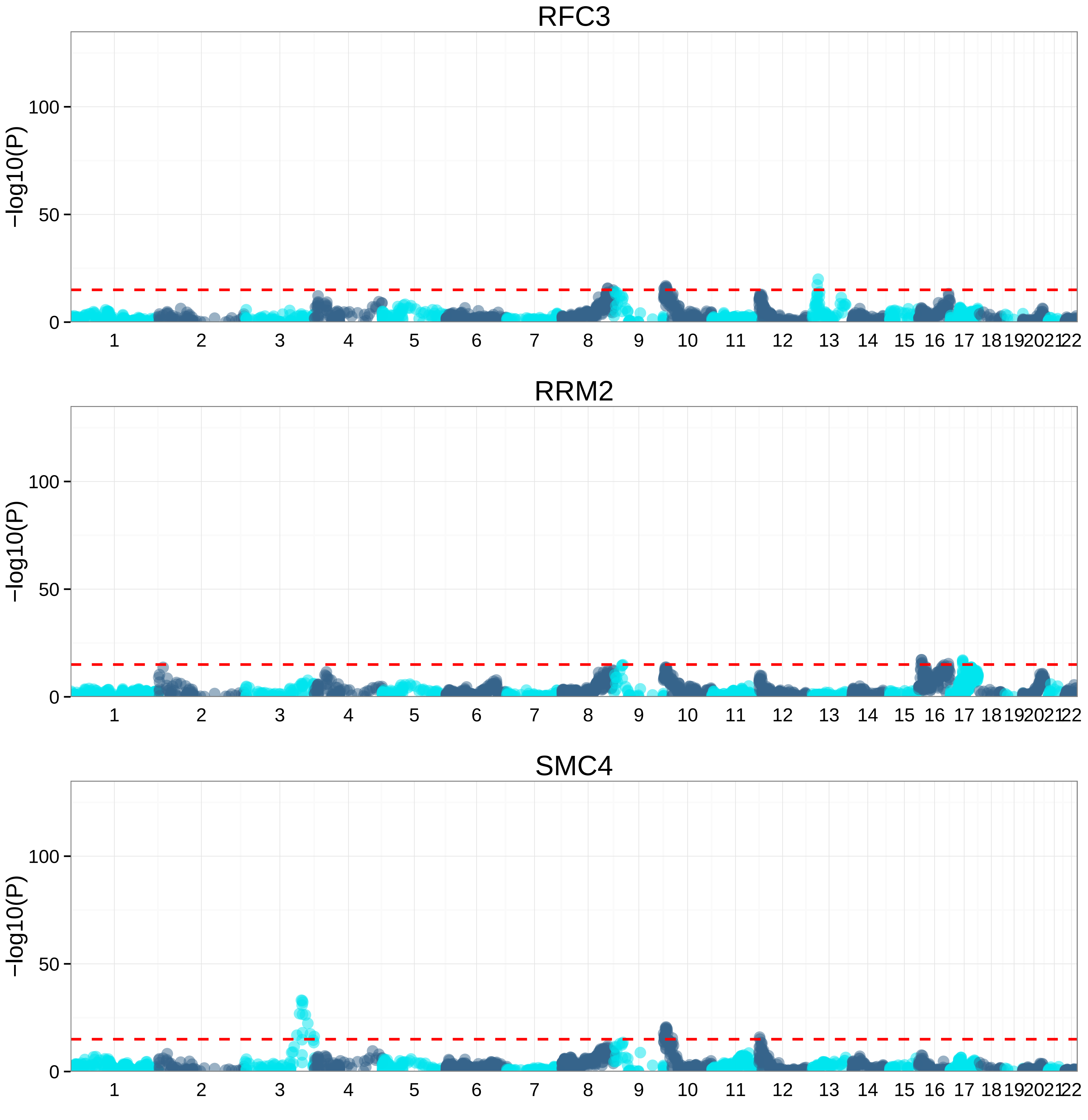
RFC3
−log10(P)
100
50
0
1
2
3
4
5
6
7
8
9
10
11
12
13
14
15
16
17
18
19
20
21
22
RRM2
−log10(P)
100
50
0
1
2
3
4
5
6
7
8
9
10
11
12
13
14
15
16
17
18
19
20
21
22
SMC4
−log10(P)
100
50
0
1
2
3
4
5
6
7
8
9
10
11
12
13
14
15
16
17
18
19
20
21
22

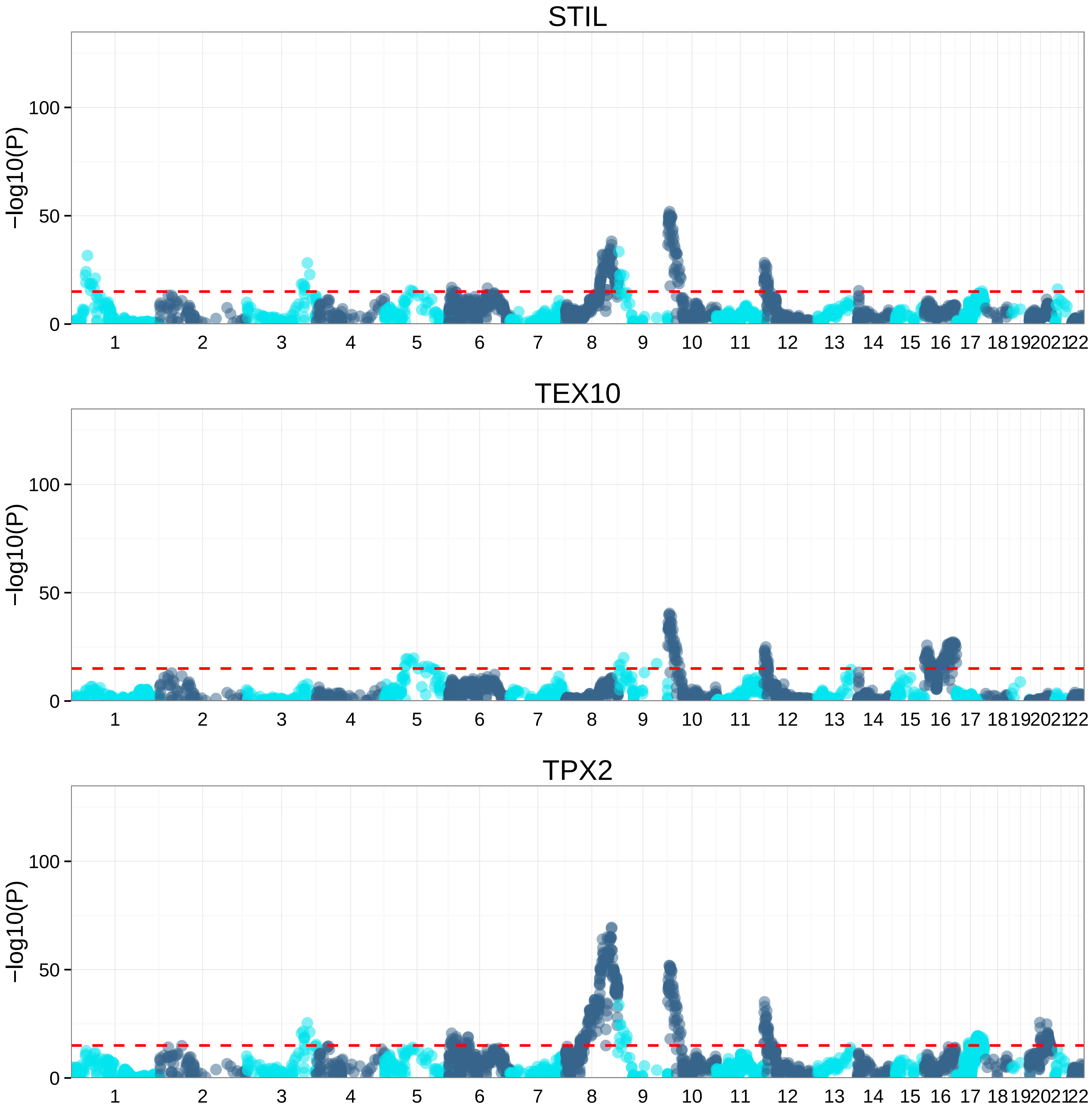

STIL
TEX10
TPX2
−log10(P)
0
50
100
1
2
3
4
5
6
7
8
9
10
11
12
13
14
15
16
17
18
19
20
21
22

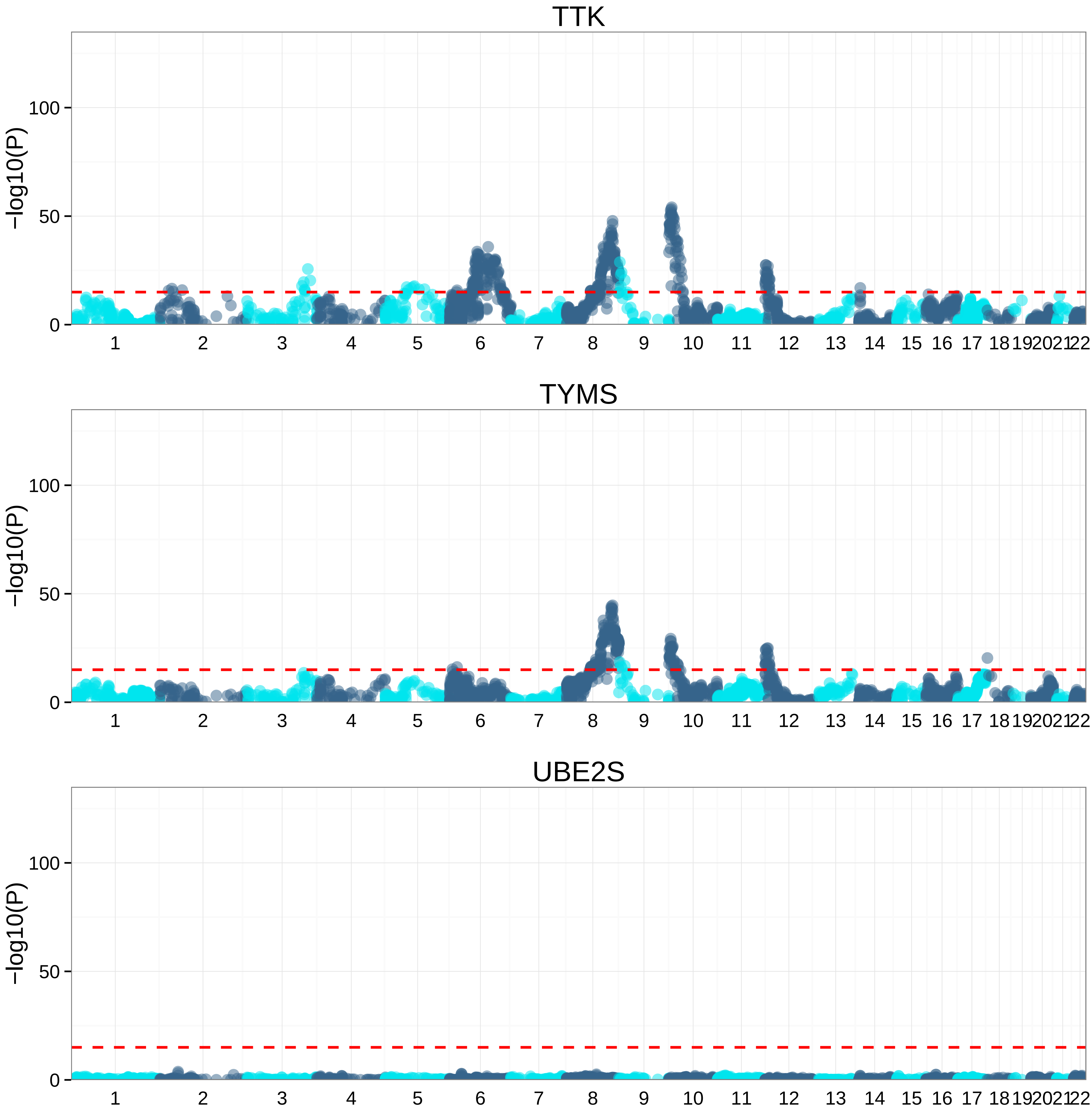

TTK
TYMS
UBE2S
−log10(P)
100
50
0
1
2
3
4
5
6
7
8
9
10
11
12
13
14
15
16
17
18
19
20
21
22
